# Supplementary material for: SFPQ directs histone H3.3 deposition to R-loops in DNA repeats to protect genome stability
Source: Nat Commun. 2026 Feb 24;17:3151. doi: 10.1038/s41467-026-69479-w (PMC13043726; doi:10.1038/s41467-026-69479-w)
Supplement: Supplementary file 1 — Supplementary Information [file 41467_2026_69479_MOESM1_ESM.pdf]

SFPQ Directs Histone H3.3 Deposition to R-Loops in DNA Repeats to Protect Genome Stability

Alessandro Ferrando[1, 6, #], Michele Giaquinto[1, #], Luisa M.R. Napolitano[2], Giulia Canarutto[1, 3], Alessandro Framarini[1], Alice Gambelli[1], Pamela Veneziano Broccia[1, 4], Annie Zappone[1], Eleonora Petti[1, 7], Chiara Boncristiani[1], Andrea Parlante[1, 3], Silvia Onesti[2], Silvano Piazza[1, 3], Roberta Benetti[5, $], Stefan Schoeftner[1, $]

1. Dipartimento di Scienze della Vita, Università degli Studi di Trieste, Via E. Weiss 2, 34127 Trieste, Italy.
2. Structural Biology Laboratory, Elettra - Sincrotrone Trieste S.C.p.A, Area Science Park Basovizza, 34149 Trieste, Italy.
3. Computational Biology, International Centre for Genetic Engineering and Biotechnology, 34149 Trieste, Italy.
4. current address, Centro Nacional de Análisis Genómico (CNAG), c/ Baldiri Reixac, 4, Barcelona Science Park - Tower I 08028 Barcelona, Spain.
5. Laboratory of Epigenomics, Department of Medicine, Università degli Studi di Udine, Udine, Italy.
6. current address, Dipartimento di Scienze Cliniche e Biologiche, Università degli Studi di Torino, Regione Gonzole, 10, 10043 Orbassano, Italy
7. current address, IRCCS - Regina Elena National Cancer Institute, Translational Oncology Research Unit, Via Elio Chianesi 53, 00144, Rome, Italy.

# These authors contributed equally to the manuscript

$ Correspondence to: roberta.benetti@uniud.it; sschoeftner@units.it

CONTENTS:

Supplementary figures

Supplementary figure 1: related to main figure 1; Title: Suppression of R-loops and replication stress by SFPQ.

Supplementary figure 2: related to main figure 2; Title: SFPQ *in vitro* binding specificity for nucleic acid substrates.

Supplementary figure 3: related to main figure 3; Title: SFPQ controls DAXX localization and ATRX expression.

Supplementary figure 4: related to main figure 4; Title: SFPQ controls chromatin organization.

Supplementary figure 5: related to main figure 5; Title: Identification of functionally relevant SFPQ domains by deletion analysis.

Supplementary figure 6: related to main figure 6; Title: SFPQ suppresses replication stress and genome instability.

Supplementary figure 7: related to main figure 7; Title: Suppression of R-loops prevents the activation of innate immunity via the cGAS/STING pathway in sarcoma.

Supplementary figure 8: related to main figure 7; Title: Expression of individual signature genes and human sarcoma patient overall survival.

Supplementary figure 9: related to main figure 7; Title: Impact of SFPQ and individual signature genes on human sarcoma patient overall survival.

Supplementary tables:

Supplementary table 1: Title: Signature genes used for Kaplan-Meier analysis in Supplementary Figure 8

Supplementary table 2: Title: Signature genes used for Kaplan-Meier analysis in Supplementary Figure 9

Supplementary table 3 Title: Synthetic siRNAs used in this study.

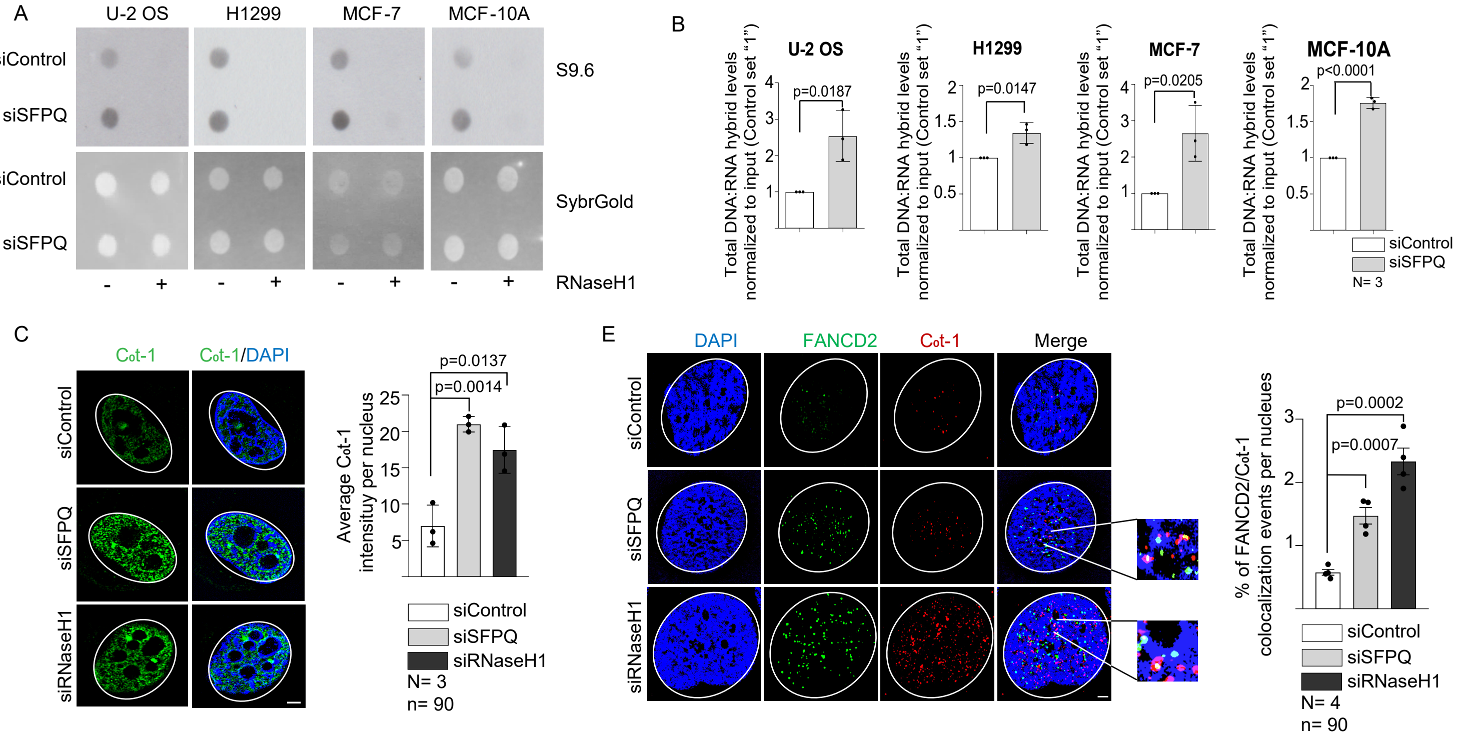

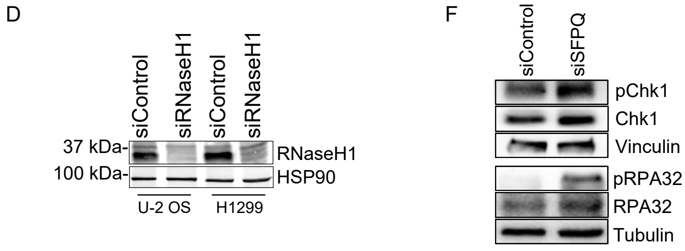

G

U-2 OS			
	Average± SD (n=3)	Average± SD (n=3)	p-value
**Cell cycle phase**	**siControl (%)**	**siSFPQ (%)**	**siCon vs. siSFPQ**
**subG1 (apoptosis)**	1.223±0.044	2.937±0.206	**0.0002**
**G0/G1**	36.57±2.857	37.1±3.200	n.s.
**S**	19.57±3.412	16.83±4.534	n.s.
**G2/M**	17.43±0.718	22.87±1.504	**0.0048**
**>G2**	24±3.377	19.27±4.565	n.s.

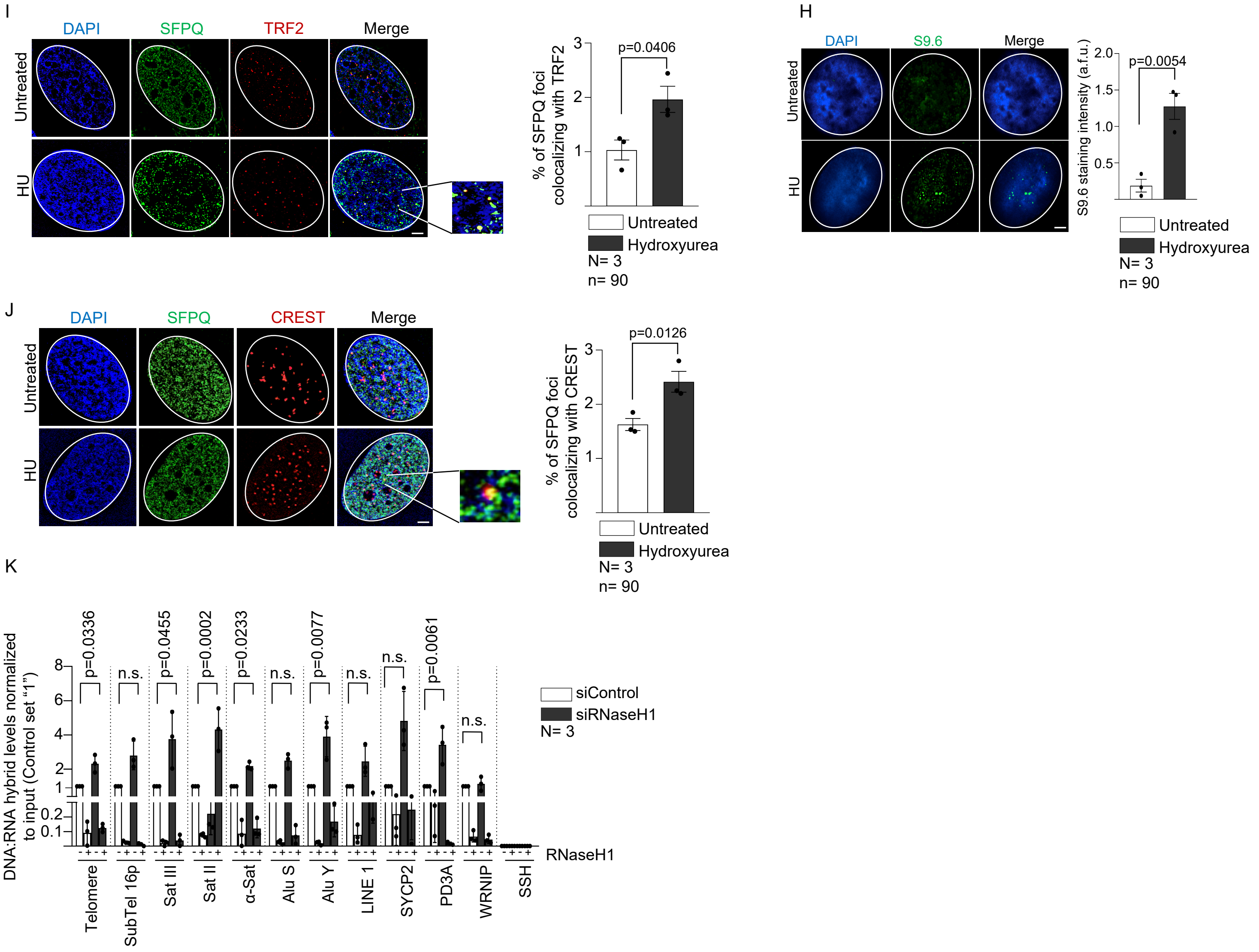

Supplementary Figure 1

(a) DNA dot-blot experiment using cancer cell lines transfected with indicated siRNAs. Representative images are shown. Prior to loading, control samples were treated with recombinant RNaseH1 to eliminate RNA:DNA hybrids. RNA:DNA hybrids were stained using a monoclonal S9.6 antibodies. As loading control, total DNA was stained using SybrGold.

(b) Quantifications of dot-blot experiments shown in (a).

(c) C_0t-1 RNA-FISH performed in H1299 cells transiently transfected with indicated siRNAs (left panel). Quantification of C_0t-1 signal intensity (right panel) as obtained by super-resolution microscopy.

(d) Western blotting analysis using the indicated antibodies to ensure RNaseH1 knockdown efficiency in both U-2 OS and H1299 cell lines.

(e) C_0t-1 RNA-FISH combined with anti-FANCD2 immunostaining performed on U-2 OS cells transfected with indicated siRNAs. Images were obtained by super-resolution microscopy.
Selected co-localization events are shown as zoomed image. Left panels, representative images. Right panel, quantification of co-localization events per nucleus

(f) Western blotting analysis using the indicated antibodies. Vinculin and alpha-tubulin specific antibodies were used as loading control.

(g) Cell cycle flow cytometry analysis of U-2 OS cells transiently transfected with control siRNAs and siRNAs targeting SFPQ.

(h) Representative image demonstrating increased R-loop levels in hydroxyurea treated U-2 OS cells. In control experiments fixed cells were treated with recombinant RNaseH1 prior to immunofluorescence (not shown). For additional quality controls related to the S9.6 monoclonal antibody, see Supplementary Figure 3e.

(i) Representative images of combined immunofluorescence with anti-SFPQ and anti-TRF2 antibodies in U-2 OS cells treated with hydroxyurea (HU) or left untreated. Images were obtained by super-resolution microscopy. Right panel, quantification of co-localization events.

(j) Combined immunofluorescence with anti-SFPQ and anti-CREST (centromere) antibodies in U-2 OS cells treated with hydroxyurea (HU) or left untreated. Right panel, quantification of co-localization events.

(k) DRIP-qPCR analysis using U-2 OS cells transiently transfected with the indicated siRNAs. Target regions for quantitative PCR analysis are indicated. *SYCP2* and *PD3A* were used as positive control for DAXX and SFPQ, respectively. *WRNIP* and *SSH* were used as negative controls. RNaseH1, treatment of genomic DNA with recombinant RNaseH1 prior to DRIP. Bars represent mean values, error bars indicate standard deviation. N, number of independent experiments. n, number of analysed nuclei. An unpaired, two-sided Student's t-test was used to calculate statistical significance; p-values are shown. Scale bar (1 µm) applies to all images in respective immunofluorescence panels. DNA was stained using DAPI (4',6-diamidino-2-phenylindole). Source data are provided with this paper.

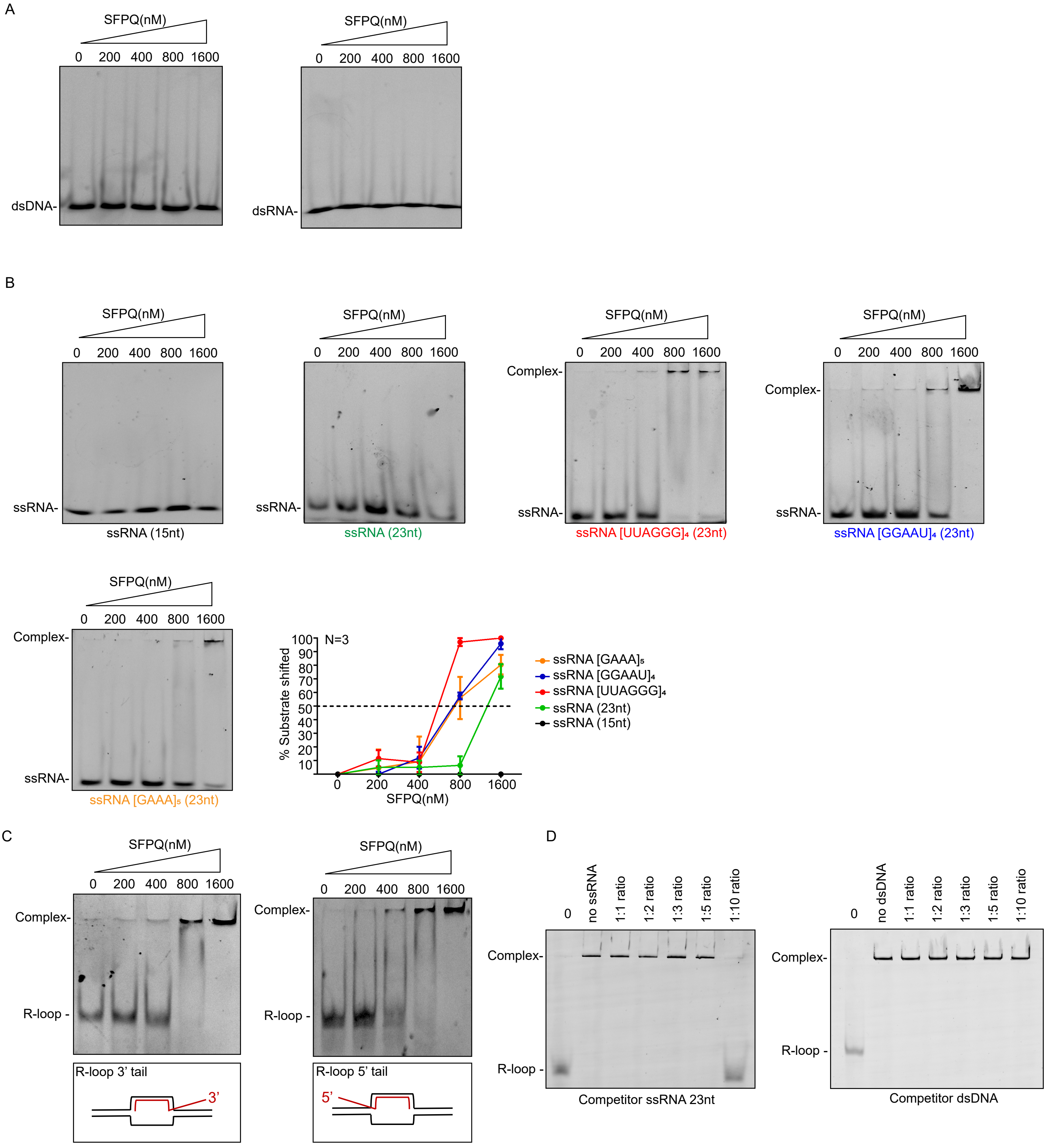
A
SFPQ(nM)
0 200 400 800 1600
dsDNA-
SFPQ(nM)
0 200 400 800 1600
dsRNA-
B
SFPQ(nM)
0 200 400 800 1600
ssRNA-
ssRNA (15nt)
SFPQ(nM)
0 200 400 800 1600
ssRNA-
ssRNA (23nt)
SFPQ(nM)
0 200 400 800 1600
Complex-
ssRNA-
ssRNA [UUAGGG]4 (23nt)
SFPQ(nM)
0 200 400 800 1600
Complex-
ssRNA-
ssRNA [GGAAU]4 (23nt)
SFPQ(nM)
0 200 400 800 1600
Complex-
ssRNA-
ssRNA [GAAA]5 (23nt)
N=3
% Substrate shifted
100 90 80 70 60 50 40 30 20 10
0 200 400 800 1600
SFPQ(nM)
ssRNA [GAAA]5
ssRNA [GGAAU]4
ssRNA [UUAGGG]4
ssRNA (23nt)
ssRNA (15nt)
C
SFPQ(nM)
0 200 400 800 1600
Complex-
R-loop -
R-loop 3' tail
3'
SFPQ(nM)
0 200 400 800 1600
Complex-
R-loop -
R-loop 5' tail
5'
D
0
no ssRNA
1:1 ratio
1:2 ratio
1:3 ratio
1:5 ratio
1:10 ratio
Complex-
R-loop -
Competitor ssRNA 23nt
0
no dsDNA
1:1 ratio
1:2 ratio
1:3 ratio
1:5 ratio
1:10 ratio
Complex-
R-loop -
Competitor dsDNA

Supplementary Figure 2

(a) EMSA using increasing amounts of recombinant SFPQ and dsDNA or dsRNA as nucleic acid substrates.

(b) EMSA using recombinant SFPQ and the indicated synthetic ssRNAs. Graph shows the percentage of substrates shifted in EMSA experiments.

(c) EMSA showing binding of recombinant SFPQ to synthetic R-loops containing protruding 3' RNA terminus (left) or 5' RNA terminus (right). Error bars indicate standard deviation; N, number of independent experiments.

(d) EMSA experiments using labelled R-loop structures and unlabelled competing ssRNA (left panel) or dsDNA (right panel); “0” no recombinant SFPQ added to probe. Molar excess ratio of competitor nucleic acids is indicated. Data in graph are represented as mean values; error bars indicate standard deviation; N= number of independent experiments. Source data are provided with this paper.

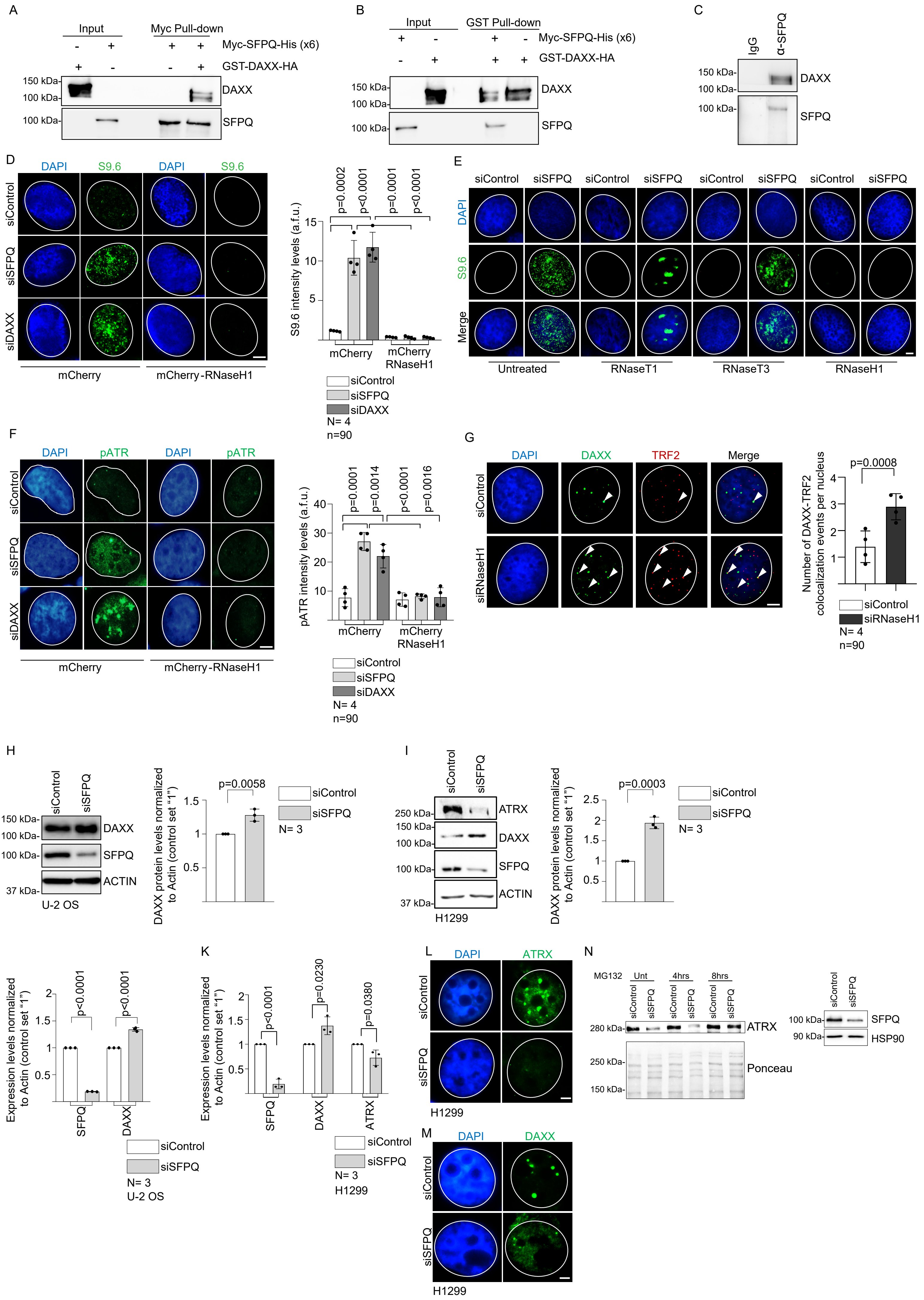

A
Input
Myc Pull-down
Myc-SFPQ-His (x6)
GST-DAXX-HA
150 kDa
100 kDa
DAXX
SFPQ
B
Input
GST Pull-down
Myc-SFPQ-His (x6)
GST-DAXX-HA
DAXX
SFPQ
C
IgG
α-SFPQ
DAXX
SFPQ
D
DAPI
S9.6
siControl
siSFPQ
siDAXX
mCherry
mCherry-RNaseH1
S9.6 intensity levels (a.f.u.)
p=0.0002
p<0.0001
p=0.0001
p<0.0001
mCherry RNaseH1
siControl
siSFPQ
siDAXX
N= 4
n=90
E
DAPI
S9.6
Merge
Untreated
RNaseT1
RNaseT3
RNaseH1
F
pATR
pATR intensity levels (a.f.u.)
p=0.0001
p=0.0014
p<0.0001
p=0.0016
G
DAXX
TRF2
Merge
siRNaseH1
Number of DAXX-TRF2 colocalization events per nucleus
p=0.0008
H
U-2 OS
ACTIN
37 kDa
DAXX protein levels normalized to Actin (control set "1")
p=0.0058
N= 3
I
H1299
ATRX
250 kDa
p=0.0003
J
Expression levels normalized to Actin (control set "1")
p<0.0001
K
p=0.0230
p=0.0380
ATRX
L
ATRX
M
N
MG132
Unt
4hrs
8hrs
280 kDa
Ponceau
250 kDa
150 kDa
90 kDa
HSP90

Supplementary Figure 3
(a) Protein pull-down assay using recombinant His6-myc-SFPQ and GST-DAXX-HA using anti-myc antibody coupled beads. Immunoblots were performed using anti-SFPQ and anti-DAXX antibodies.
(b) Protein pull-down assay using recombinant His6-Myc-SFPQ and GST-DAXX-HA using glutathione coupled beads. Immunoblots were performed using anti-SFPQ and anti-DAXX antibodies.
(c) Western blotting of reverse crosslinked proteins obtained from ChIP using antibodies specific for endogenous SFPQ or control IgG. Immunoblots were performed using anti-SFPQ and anti-DAXX antibodies.
(d) Immunofluorescence staining using monoclonal anti-RNA:DNA hybrid (S9.6) antibodies in U-2 OS cells expressing mCherry or mCherry-RNaseH1 cells after transfection with indicated siRNAs. Left panels, representative images. Right panel, quantification of pan-nuclear immunofluorescence intensity.
(e) Validation of substrate specificity of S9.6 monoclonal antibodies. Fixed cells were pre-treated with the indicated recombinant enzymes before proceeding with anti-RNA:DNA hybrid immunofluorescence. Only RNaseH1 treatment completely eliminated RNA:DNA hybrid signals.
(f) Immunofluorescence analysis using anti-p-ATR (Thr1989) antibodies on U-2 OS cells expressing mCherry and mCherry-RNaseH1 after transfection with indicated siRNAs. Left panel, representative images. Right panel, quantification of pan-nuclear immunofluorescence intensity.
(g) Combined immunofluorescence using anti-DAXX and anti-TRF2 antibodies on U-2 OS cells transfected with indicated siRNAs. Left panel, representative images, arrowheads indicate co-localization events. Right panel, quantification of co-localization events per nucleus.
(h) Western blotting using whole cells extracts from U-2 OS cells transiently transfected with the indicated siRNAs. Anti-DAXX, anti-SFPQ, and anti-ACTIN specific antibodies were used in western blotting experiments. Right panel, quantification of DAXX protein levels.
(i) Western blotting using whole cells extracts from H1299 cells transiently transfected with the indicated siRNAs. Anti-ATRX, anti-DAXX, anti-SFPQ, and anti-ACTIN specific antibodies were used in western blotting experiments. Right panel, quantification of DAXX protein levels.
(j, k) Quantitative real-time PCR on the indicated genes in U-2 OS (j) or H1299 (k) transfected with control and SFPQ specific siRNAs. Actin was used as reference gene.
(l) Representative images of immunofluorescence analysis using anti-ATRX antibodies. H1299 cells were transiently transfected with indicated siRNAs.
(m) Representative images of immunofluorescence analysis using anti-DAXX antibodies. H1299 cells were transiently transfected with indicated siRNAs.
(n) Western blotting of whole cell extracts of H1299 cells transiently transfected with control or SFPQ specific siRNAs. Prior to lysis, cells were treated with MG132 for the indicated time periods (left panel). Right panel, confirmation of SFPQ knock-down using lysates collected prior to MG132 treatment.
In quantification blots, bars represent mean values, error bars indicate standard deviation. N, number of independent experiments. n, number of analysed nuclei. An unpaired, two-sided Student's t-test was used to calculate statistical significance; p-values are shown. Scale bar (1 µm) applies to all images in respective immunofluorescence panels. DNA was stained using DAPI (4',6-diamidino-2-phenylindole). siRNAs are listed in Supplementary Table 3. Source data are provided with this paper.

A

	SFPQ	DAXX	H3.3
siControl	6375	6643	29869
siSFPQ	//	2816	55388

B

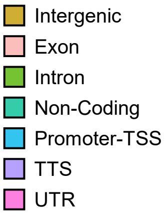

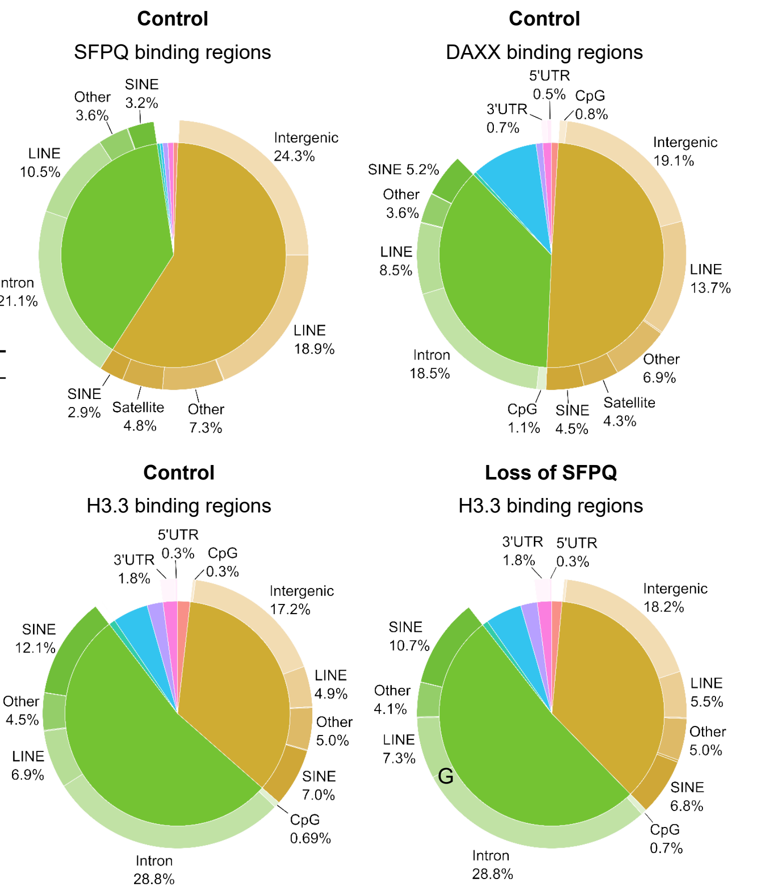

C

SFPQ binding regions (%)

Region	siCon
3'UTR	0.80
5'UTR	0.02
CpG	0.09
Exon	0.61
Intergenic	24.30
Intron	21.16
LINE	29.35
Non-Coding	0.41
Other	8.82
Promoter-TSS	0.39
Satellite	4.96
Simple Repeat	2.26
SINE	6.10
TTS	0.74

DAXX binding regions (%)

Region	siCon	siSFPQ
3'UTR	0.72	0.50
5'UTR	0.54	1.59
CpG	1.99	5.34
Exon	0.96	2.45
Intergenic	19.10	15.07
Intron	18.53	14.85
LINE	22.29	11.61
Non-Coding	0.50	0.94
Other	7.77	6.71
Promoter-TSS	9.54	21.12
Satellite	4.34	7.35
Simple Repeat	3.04	3.68
SINE	9.71	7.64
TTS	0.96	1.15

H3.3 binding regions (%)

Region	siCon	siSFPQ
3'UTR	1.77	1.77
5'UTR	0.32	0.32
CpG	1.02	1.02
Exon	1.46	1.80
Intergenic	18.14	17.20
Intron	28.77	28.79
LINE	12.86	11.80
Non-Coding	0.82	0.93
Other	8.33	8.57
Promoter-TSS	5.24	5.07
Satellite	0.29	0.13
Simple Repeat	1.08	1.17
SINE	17.52	19.14
TTS	2.38	2.29

D

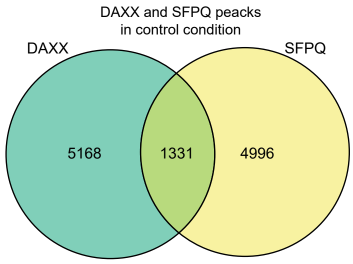

E

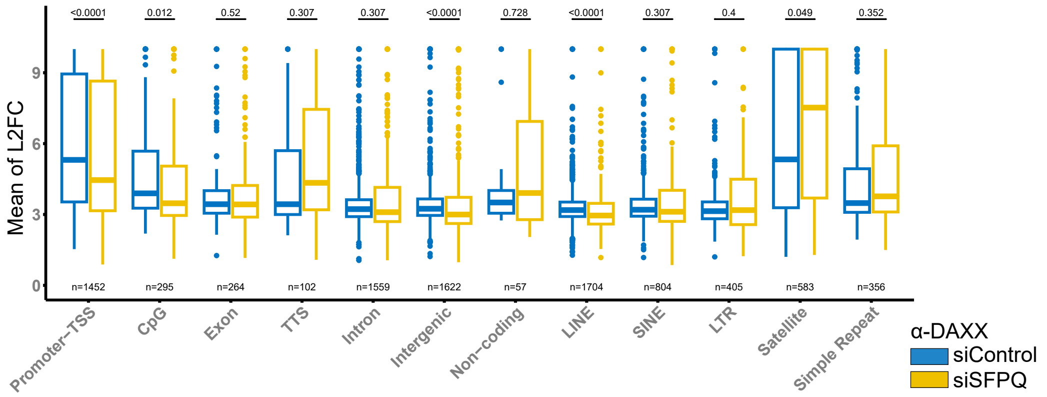

F

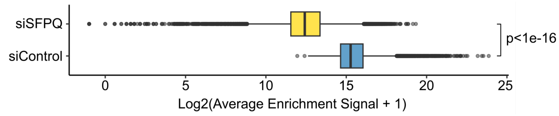

G

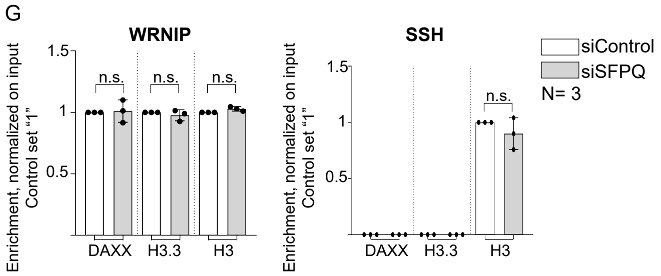

H

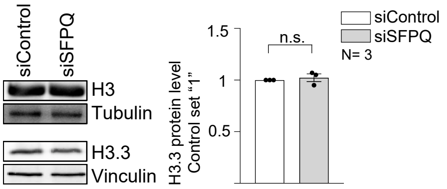

Supplementary Figure 4
(a) Table reporting the number of peaks (e.g. binding sites) of indicated proteins obtained by ChiP-Seq of indicated experimental conditions. Control, U-2 OS cells transiently transfected with control siRNAs; loss of SFPQ, U-2 OS cells transiently transfected with SFPQ specific siRNAs.
(b) Pie charts representing the distribution of SFPQ (top left panel), DAXX (top right panel), or H3.3 (bottom left panel) peaks across the indicated sequence categories after ChIP-Seq. Chromatin from control siRNA transfected U-2 OS cells was used for the analysis. Bottom right panel, ChIP using chromatin from U-2 OS cells transiently transfected with SFPQ specific siRNAs; distribution of H3.3 peaks across different sequence categories is shown.
(c) Table reporting the percentage of binding sites of indicated proteins at listed genome regions. Chromatin from control siRNA (siCon) and SFPQ specific siRNAs (siSFPQ) transfected U-2 OS cells was used.
(d) Venn diagram representing the number of unique or common SFPQ and DAXX peaks obtained by ChIP seq using chromatin from U-2 OS cells transiently transfected with control and SFPQ specific siRNAs.
(e) Box-plot representing DAXX mean Log2 Fold Change at reported genomic regions in control (blue) and SFPQ depleted (yellow) U-2 OS cells. n, number of peaks for reported genomic region for both conditions. P-values were calculated with Wilcox test and adjusted for False Discovery Rate. Center, median (50th percentile); box bounds, Q1 (25th percentile) and Q3 (75th percentile); whiskers, minimum and maximum non-outlier values (within 1.5×IQR of Q1/Q3); dots, outliers. Promoter-TSS = Promoter-Transcription Starting Site; CpG = CpG islands; TTS = Transcription Termination Site; LINE = Long Interspersed Nuclear Element; SINE = Short Interspersed Nuclear Elements; LTR = Long Terminal Repeat.
(f) Box plot representing area under the curve (AUC) calculation for individual H3.3 peaks in control and SFPQ depleted U-2 OS cells. p-value was calculated using the Wilcoxon rank sum test with continuity correction. Center, median (50th percentile); box bounds, Q1 (25th percentile) and Q3 (75th percentile); whiskers, minimum and maximum non-outlier values (within 1.5×IQR of Q1/Q3); dots, outliers.
(g) Chromatin immunoprecipitation (ChIP) assay performed on U-2 OS cells transiently transfected with indicated siRNAs. The graph reports the binding of DAXX, H3.3, and H3 to negative control regions in *WRNIP* (left panel) and *SSH* genes (right panel). Alterations are shown as fold-change between control and SFPQ knock-down U-2 OS cells.
(h) Western blotting of U-2 OS cells transiently transfected with the indicated siRNAs. Loss of SFPQ does not impact on H3.3 or H3 protein levels.
In quantification blots g and h, bars represent the mean values, error bars indicate standard deviation. N= number of independent experiments. An unpaired, two-sided Student’s t-test was used to calculate statistical significance; p-values are shown. Source data are provided with this paper.

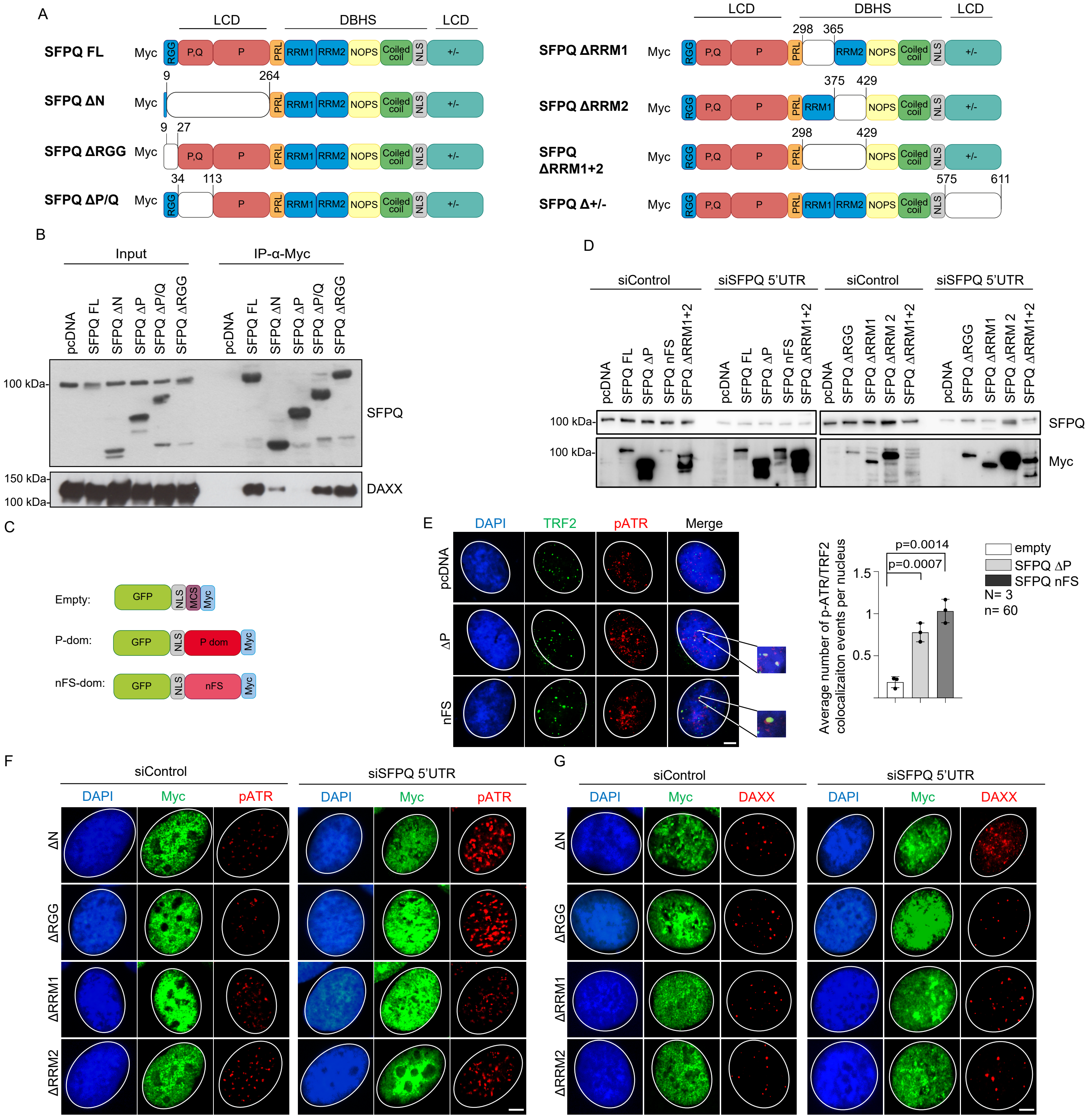
A
LCD
DBHS
LCD
SFPQ FL
Myc
RGG
P,Q
P
PRL
RRM1
RRM2
NOPS
Coiled coil
NLS
+/-
SFPQ ΔN
9
264
SFPQ ΔRGG
9
27
SFPQ ΔP/Q
34
113
SFPQ ΔRRM1
298
365
SFPQ ΔRRM2
375
429
SFPQ ΔRRM1+2
298
429
SFPQ Δ+/-
575
611
B
Input
IP-α-Myc
pcDNA
SFPQ FL
SFPQ ΔN
SFPQ ΔP
SFPQ ΔP/Q
SFPQ ΔRGG
100 kDa-
SFPQ
150 kDa-
100 kDa-
DAXX
C
Empty:
GFP
NLS
MCS
Myc
P-dom:
P dom
nFS-dom:
nFS
D
siControl
siSFPQ 5'UTR
SFPQ nFS
SFPQ ΔRRM1+2
SFPQ ΔRRM1
SFPQ ΔRRM 2
E
DAPI
TRF2
pATR
Merge
ΔP
nFS
Average number of p-ATR/TRF2 colocalizaiton events per nucleus
p=0.0014
p=0.0007
1.5
1
0.5
empty
SFPQ ΔP
SFPQ nFS
N= 3
n= 60
F
Myc
ΔRGG
ΔRRM1
ΔRRM2
G
DAXX

Supplementary Figure 5

(a) Schematic representation of SFPQ domains and mutants. Full-length (FL) SFPQ is composed of the listed domains: arginine-glycine-glycine (RGG) box (blue); N- and C-terminal low-complexity domains (LCDs); proline/glutamine-rich subdomain (P,Q, red); proline-rich domain (P, red) ; PR linker (PRL, orange); the DBHS conserved region, containing RNA-recognition motifs (RRM1 and RRM2, blue), NonA/paraspeckles (NOPS) domain (yellow), the coiled-coil domain (green), a nuclear-localization sequence (NLS) (in grey) and the +/- domain (bright green). Domains that were deleted in the individual expression constructs are shown as white boxes. Numbers indicate amino acid positions in full-length SFPQ.

(b) Immunoprecipitation experiments using U-2 OS cells transiently co-transfected with an expression vector for full-length, HA-tagged DAXX and a control vector (pcDNA) or expression vectors encoding the indicated, myc-tagged SFPQ versions (FL, full length). Immunoblots were performed using anti-myc and anti-HA antibodies.

(c) Schematic representation of expression vectors encoding for the myc-tagged SFPQ P-domain or nFS-domain, fused with a nuclear localization signal (NLS) and a GFP open reading frame.

(d) Immunoblot performed using whole cell extracts from U-2 OS cells stably expressing myc-tagged full length (FL) or listed SFPQ mutants that were transiently transfected with control and SFPQ specific siRNAs. Western blotting was performed using the indicated antibodies.

(e) Colocalization of TRF2 and phosphorylated ATR (Thr1989) in U-2 OS cells transiently overexpressing dominant negative SFPQ (SFPQ dP and SFPQ nFS, left panel). Right panel, quantification of immunofluorescence data.

(f) Immunofluorescence analysis of U-2 OS cells stably expressing myc-tagged SFPQ mutants after transient transfection with control siRNAs or siRNAs specifically targeting the 5'UTR of endogenous SFPQ. Anti-myc and anti-p-ATR(Thr1989) specific antibodies were used for immunodetection. Representative images are shown. Experiments were carried out in parallel with data shown in Fig. 5e. Quantification is shown in Fig. 5f and is limited to myc-positive cells.

(g) Immunofluorescence analysis of U-2 OS cells stably expressing myc-tagged SFPQ mutants after transient transfection with control siRNAs or siRNAs specifically targeting the 5'UTR of endogenous SFPQ. Anti-myc and anti-DAXX specific antibodies were used for immunodetection. Representative images are shown. Experiments were carried out in parallel with data shown in Fig. 5g.

(e-g) For quantifications, data represents the mean, error bars indicate standard deviation. N= number of independent experiments. n= number of analysed nuclei. An unpaired, two-sided Student's t-test was used to calculate statistical significance. Scale bar (1 µm) applies to all images in respective immunofluorescence panels. DNA was stained using DAPI (4',6-diamidino-2-phenylindole). Source data are provided with this paper.

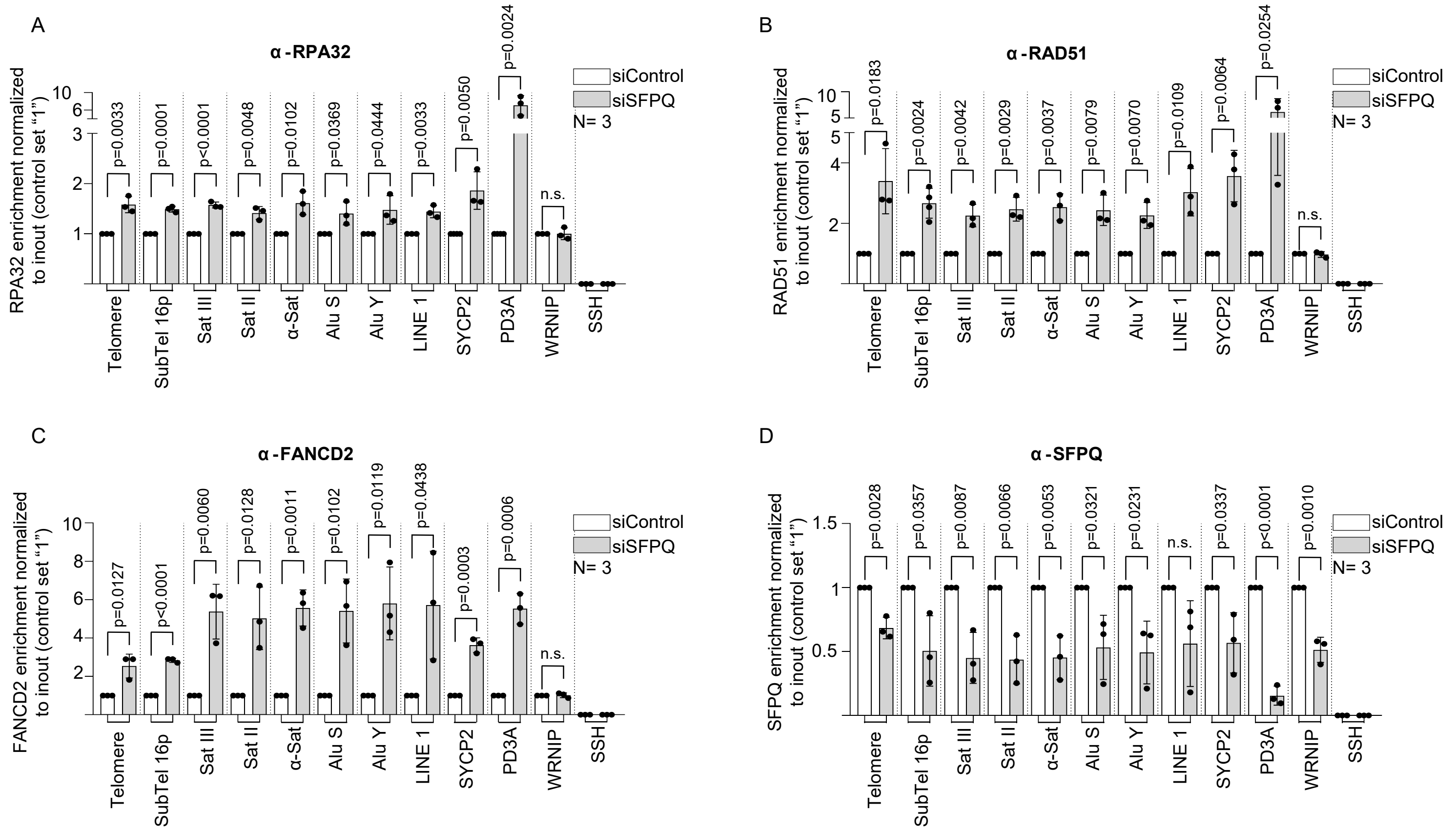

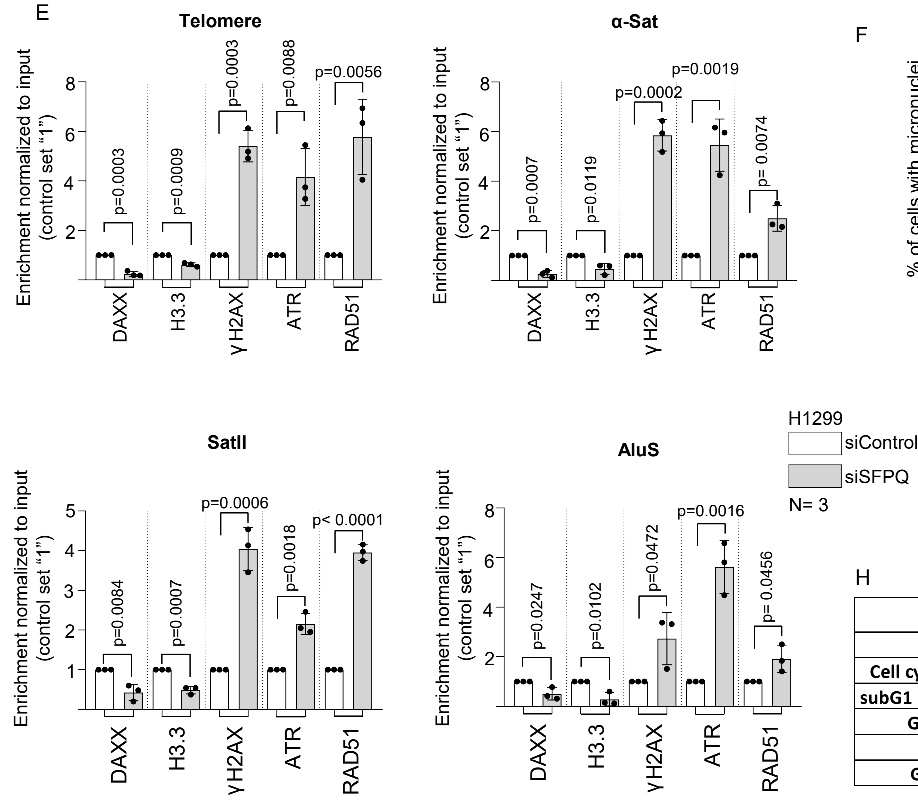

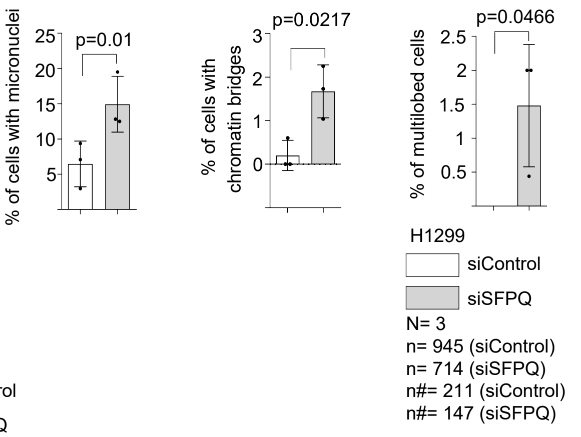

H

MCF-10A			
	Average± SD (N=3)	Average± SD (N=3)	p-value
**Cell cycle phase**	**siControl (%)**	**siSFPQ (%)**	**siCon vs. siSFPQ**
**subG1 (apoptosis)**	0.84±0.088	10.51±1.516	**0.0004**
**G0/G1**	79.9±1.087	56.25±1.732	**<0.0001**
**S**	11.293±0.497	19.78±2.452	**0.0042**
**G2/M**	2.32±0.252	7.06±1.539	**0.0062**

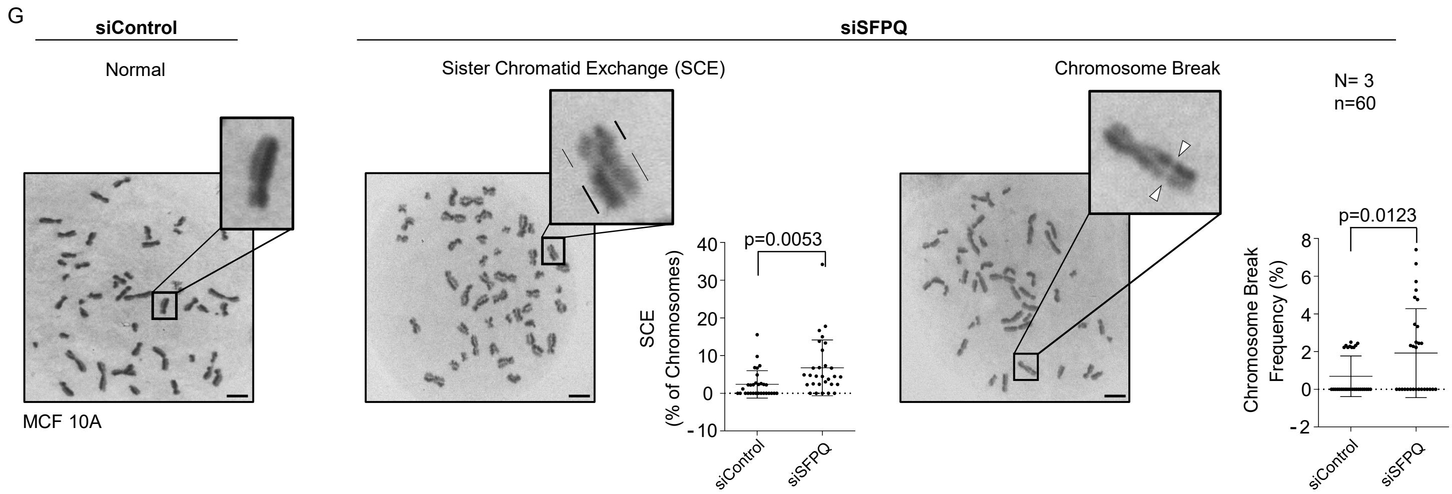

Supplementary Figure 6
(a - d) Chromatin immunoprecipitation (ChIP) assay experiments performed on U-2 OS cells transiently transfected with control siRNAs and siRNAs targeting SFPQ. Quantitative real-time PCR was carried out using specific primer pairs for the indicated genome regions. *PD3A* and *WRNIP* were used as positive control for SFPQ and DAXX binding, respectively. *SSH* was used as negative control. Graphs reports the binding of RPA32 (a), RAD51 (b), FANCD2 (c), and SFPQ (d) to the indicated regions. Fold change values were calculated using control siRNA transfected cells as reference. Amplification values from ChIP using IgG from pre-immune serum was subtracted during normalization.
(e) Chromatin immunoprecipitation (ChIP) assay using chromatin from H1299 cells transiently transfected using control siRNAs and siRNAs targeting SFPQ. Graphs report the binding of DAXX, H3.3, γH2AX, ATR, and RAD51 to the indicated genome region of interest, as detected by quantitative real-time PCR. Enrichment is expressed as fold-change values normalize to control siRNA transfected H1299 cells. Amplification values from ChIP using IgG from pre-immune serum was subtracted during normalization.
(f) Quantification of time-lapse microscopy experiments using H1299 cells stably expressing GFP-tagged H2B and transiently transfected with control siRNAs and siRNAs targeting SFPQ. Frequency of micronuclei, chromatin bridges and multilobal cells are shown. n, number of analysed nuclei for the micronuclei experiment; n#= number of mitotic cells analysed for the chromatin bridges and multilobal cells.
(g) Metaphase chromosome aberrations observed in MCF 10A cells transiently transfected with control siRNAs and siRNAs targeting SFPQ. Representative Giemsa-stained, normal metaphase chromosomes (siControl) and chromosomes from SFPQ knock-down cells with increased sister chromatid exchange (SCE) rate and chromosomal breaks are shown. Fat and thin lines along chromosome indicate a representative event of sister chromatid exchange. Empty arrowheads indicate position of chromosome break. Graphs show the frequency of chromosome aberration events; n= number of analysed metaphases. Scale bar (1 μm) applies to all images. DNA was stained using Giemsa.
(h) Cell cycle flow cytometry analysis of MCF 10A cells transiently transfected with control siRNAs and siRNAs targeting SFPQ
For quantifications, bars represent mean values, error bars indicate standard deviation. N= number of independent experiments. An unpaired, two-sided Student’s t-test was used to calculate statistical significance; p-values are shown. Source data are provided with this paper.

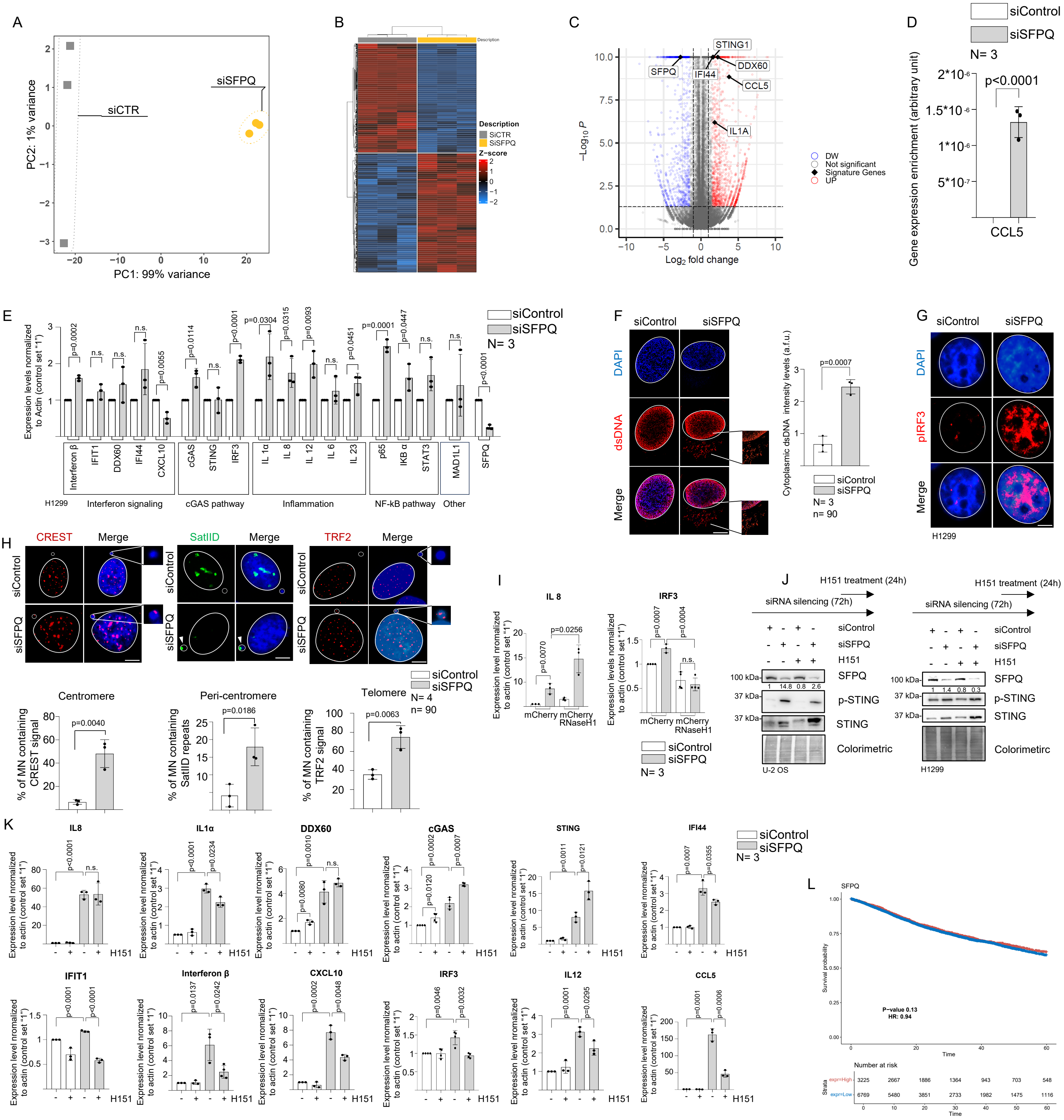

A
siSFPQ
siCTR
PC2: 1% variance
PC1: 99% variance
B
Description
SiCTR
SiSFPQ
Z-score
C
STING1
DDX60
SFPQ
IFI44
CCL5
IL1A
−Log10 P
Log2 fold change
DW
Not significant
Signature Genes
UP
D
siControl
siSFPQ
N= 3
p<0.0001
Gene expression enrichment (arbitrary unit)
CCL5
E
Expression levels normalized to Actin (control set "1")
siControl
siSFPQ
N= 3
Interferon β
IFIT1
DDX60
IFI44
CXCL10
cGAS
STING
IRF3
IL 1α
IL 8
IL 12
IL 6
IL 23
p65
IKB α
STAT3
MAD1L1
SFPQ
H1299
Interferon signaling
cGAS pathway
Inflammation
NF-kB pathway
Other
F
siControl
siSFPQ
DAPI
dsDNA
Merge
Cytoplasmic dsDNA intensity levels (a.f.u.)
p=0.0007
N= 3
n= 90
G
pIRF3
H1299
H
CREST
SatIID
TRF2
Centromere
Peri-centromere
Telomere
% of MN containing CREST signal
% of MN containing SatIID repeats
% of MN containing TRF2 signal
p=0.0040
p=0.0186
p=0.0063
N= 4
n= 90
I
IL 8
IRF3
Expression level nromalized to actin (control set "1")
mCherry
mCherry RNaseH1
J
H151 treatment (24h)
siRNA silencing (72h)
SFPQ
p-STING
STING
Colorimetric
U-2 OS
H1299
K
IL8
IL1α
DDX60
cGAS
STING
IFI44
IFIT1
Interferon β
CXCL10
IRF3
IL12
CCL5
H151
L
SFPQ
Survival probability
P-value 0.13
HR: 0.94
Time
Number at risk
Strata
expr=High
expr=Low

Supplementary Figure 7

(a) Principal Component Analysis (PCA) performed on RNA-Seq experiment using three biological replicates. U-2 OS cells were transfected with control siRNA and siRNA targeting SFPQ. PC1 shows 99% variance and PC2 1%. Yellow dots correspond to SFPQ knock-down U-2 OS cells, grey squares correspond to U-2 OS cells transfected with control siRNAs.
(b) Heatmap of Z-score of differentially expressed genes (DEGs) after siRNA mediated depletion of SFPQ from U-2 OS cells silenced with indicated siRNA. Clustering was performed using Euclidean distance and “average” as clustering method. Red, upregulated genes; blue, downregulated genes.
(c) Volcano plot representing genes that are differentially expressed in U-2 OS cells transfected with control and SFPQ specific siRNAs. Selected genes from the innate signature genes (Fig. 7b) and SFPQ are highlighted. Red, upregulated genes; blue, downregulated genes; gray, not significantly altered, black diamond, innate immunity signature gene.
(d) Quantitative RT-PCR of CCL5 in U-2 OS cells after transient siRNA mediated depletion of SFPQ
(e) Quantitative RT-PCR of indicated genes in H1299 cells transfected with control or SFPQ specific siRNAs. N, number of independent experiments. An unpaired, two-sided Student’s t-test was used to calculate statistical significance; p-values are shown.
(f) Immunofluorescence analysis using specific antibodies detecting dsDNA in the cytosol of experimental U-2 OS cells. Left panels, representative images; right panel, quantification of cytoplasmatic dsDNA signal intensity.
(g) Immunofluorescence analysis using specific antibodies for the phosphorylated version of IRF3 (pIRF3) on H1299 cells transiently transfected with control siRNAs and siRNAs targeting the SFPQ mRNA. Representative images are shown.
(h) Immunofluorescence and DNA -FISH analyses on U-2 OS cells transiently transfected with control and SFPQ specific siRNAs; representative images are shown in panels on the left. Left panels, anti-CREST; central panel, SatIID DNA FISH; right panels, anti-TRF2 antibodies. Arrowheads indicate micronuclei in representative images. Quantification of micronuclei positive for CREST antigen (bottom left), SatIID DNA (bottom central) or TRF2 (bottom right) are shown.
(i) U-2 OS cells with doxycycline inducible expression constructs for mCherry or mChery-RNaseH1 were transiently transfected with the indicated siRNAs. IL-8 and IRF3 expression was monitored by quantitative RT-PCR.
(J) Representative western-blot of U-2 OS (left) or H1299 (right) cells transfected with indicated siRNAs. Experimental cells were treated with the STING inhibitor H151 or left untreated. Western blotting was performed using the indicated antibodies.
(k) Quantitative real-time PCR using cDNA from U-2 OS cells transfected with indicated siRNAs and treated with the STING inhibitor H151, or left untreated.
(l) Kaplan-Meier plot analysis showing no correlation between SFPQ expression and patient overall survival using data from TCGA All Pancancer data. Patients were categorized based on the expression levels of SFPQ and their survival was censored at 60 months. HR is the hazard ratio calculated by the Cox regression model. HR p-value is 0.127. Patient numbers are inciated.
For quantifications, bars represent mean values, error bars indicate standard deviation. N= number of independent experiments, n= number of analyzed nucleus. An unpaired, two-sided Student’s t-test was used to calculate statistical significance. Scale bar (1 µm) applies to all images in respective immunofluorescence panels. DNA was stained using DAPI (4',6-diamidino-2-phenylindole). Source data are provided with this paper.

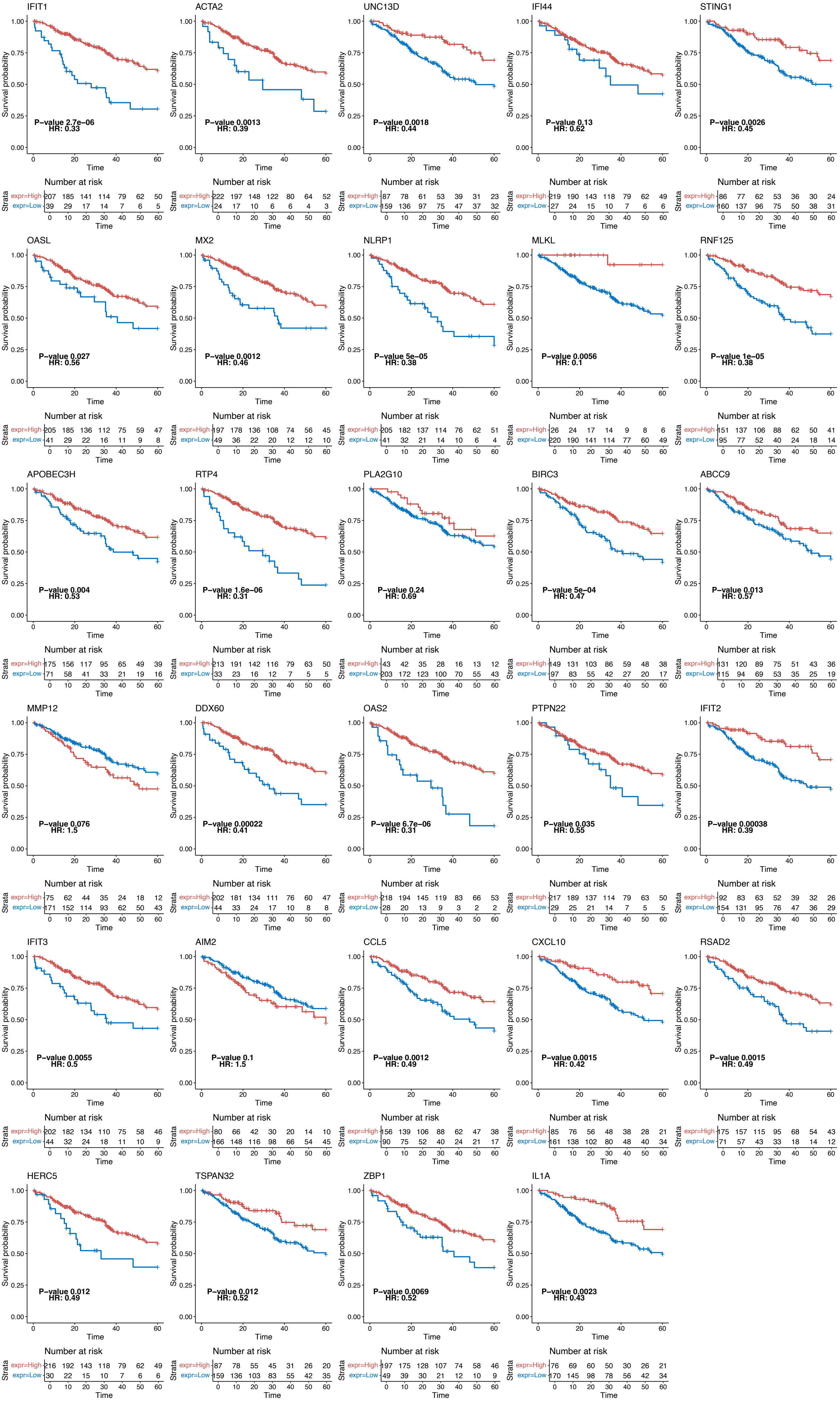

Survival probability
Time
Strata
Number at risk
IFIT1
P−value 2.7e−06
HR: 0.33
expr=High 207 185 141 114 79 62 50
expr=Low 39 29 17 14 7 6 5
ACTA2
P−value 0.0013
HR: 0.39
expr=High 222 197 148 122 80 64 52
expr=Low 24 17 10 6 6 4 3
UNC13D
P−value 0.0018
HR: 0.44
expr=High 87 78 61 53 39 31 23
expr=Low 159 136 97 75 47 37 32
IFI44
P−value 0.13
HR: 0.62
expr=High 219 190 143 118 79 62 49
expr=Low 27 24 15 10 7 6 6
STING1
P−value 0.0026
HR: 0.45
expr=High 86 77 62 53 36 30 24
expr=Low 160 137 96 75 50 38 31
OASL
P−value 0.027
HR: 0.56
expr=High 205 185 136 112 75 59 47
expr=Low 41 29 22 16 11 9 8
MX2
P−value 0.0012
HR: 0.46
expr=High 197 178 136 108 74 56 45
expr=Low 49 36 22 20 12 12 10
NLRP1
P−value 5e−05
HR: 0.38
expr=High 205 182 137 114 76 62 51
expr=Low 41 32 21 14 10 6 4
MLKL
P−value 0.0056
HR: 0.1
expr=High 26 24 17 14 9 8 6
expr=Low 220 190 141 114 77 60 49
RNF125
P−value 1e−05
HR: 0.38
expr=High 151 137 106 88 62 50 41
expr=Low 95 77 52 40 24 18 14
APOBEC3H
P−value 0.004
HR: 0.53
expr=High 175 156 117 95 65 49 39
expr=Low 71 58 41 33 21 19 16
RTP4
P−value 1.6e−06
HR: 0.31
expr=High 213 191 142 116 79 63 50
expr=Low 33 23 16 12 7 5 5
PLA2G10
P−value 0.24
HR: 0.69
expr=High 43 42 35 28 16 13 12
expr=Low 203 172 123 100 70 55 43
BIRC3
P−value 5e−04
HR: 0.47
expr=High 149 131 103 86 59 48 38
expr=Low 97 83 55 42 27 20 17
ABCC9
P−value 0.013
HR: 0.57
expr=High 131 120 89 75 51 43 36
expr=Low 115 94 69 53 35 25 19
MMP12
P−value 0.076
HR: 1.5
expr=High 75 62 44 35 24 18 12
expr=Low 171 152 114 93 62 50 43
DDX60
P−value 0.00022
HR: 0.41
expr=High 202 181 134 111 76 60 47
expr=Low 44 33 24 17 10 8 8
OAS2
P−value 6.7e−06
HR: 0.31
expr=High 218 194 145 119 83 66 53
expr=Low 28 20 13 9 3 2 2
PTPN22
P−value 0.035
HR: 0.55
expr=High 217 189 137 114 79 63 50
expr=Low 29 25 21 14 7 5 5
IFIT2
P−value 0.00038
HR: 0.39
expr=High 92 83 63 52 39 32 26
expr=Low 154 131 95 76 47 36 29
IFIT3
P−value 0.0055
HR: 0.5
expr=High 202 182 134 110 75 58 46
expr=Low 44 32 24 18 11 10 9
AIM2
P−value 0.1
HR: 1.5
expr=High 80 66 42 30 20 14 10
expr=Low 166 148 116 98 66 54 45
CCL5
P−value 0.0012
HR: 0.49
expr=High 156 139 106 88 62 47 38
expr=Low 90 75 52 40 24 21 17
CXCL10
P−value 0.0015
HR: 0.42
expr=High 85 76 56 48 38 28 21
expr=Low 161 138 102 80 48 40 34
RSAD2
P−value 0.0015
HR: 0.49
expr=High 175 157 115 95 68 54 43
expr=Low 71 57 43 33 18 14 12
HERC5
P−value 0.012
HR: 0.49
expr=High 216 192 143 118 79 62 49
expr=Low 30 22 15 10 7 6 6
TSPAN32
P−value 0.012
HR: 0.52
expr=High 87 78 55 45 31 26 20
expr=Low 159 136 103 83 55 42 35
ZBP1
P−value 0.0069
HR: 0.52
expr=High 197 175 128 107 74 58 46
expr=Low 49 39 30 21 12 10 9
IL1A
P−value 0.0023
HR: 0.43
expr=High 76 69 60 50 30 26 21
expr=Low 170 145 98 78 56 42 34

Supplementary Figure 8

Kaplan-Meier plot analysis showing correlation between higher signature genes expression and improved patient overall survival using data from TCGA pan-cancer sarcoma data set. Patients were categorized based on the expression levels of SFPQ and their survival was censored at 60 months. Hazard ratios, hazard ratios p-values and curves p-values are shown in table. SFPQ dependent, innate immunity gene expression signature genes were obtained by RNA-Seq (Figure 7b; *IFIT1, ACTA2, UNC13D, IFI44, STING1, OASL, MX2, NLRP1, MLKL, RNF125, APOBEC3H, RTP4, PLAG2G10, BIRC3, ABCC9, MMP12, DDX60, OAS2, PTPN22, IFIT2, IFIT3, AIM2, CCL5, CXCL10, RSAD2, HERC5, TSPAN32, ZBP1* and *IL1A*).

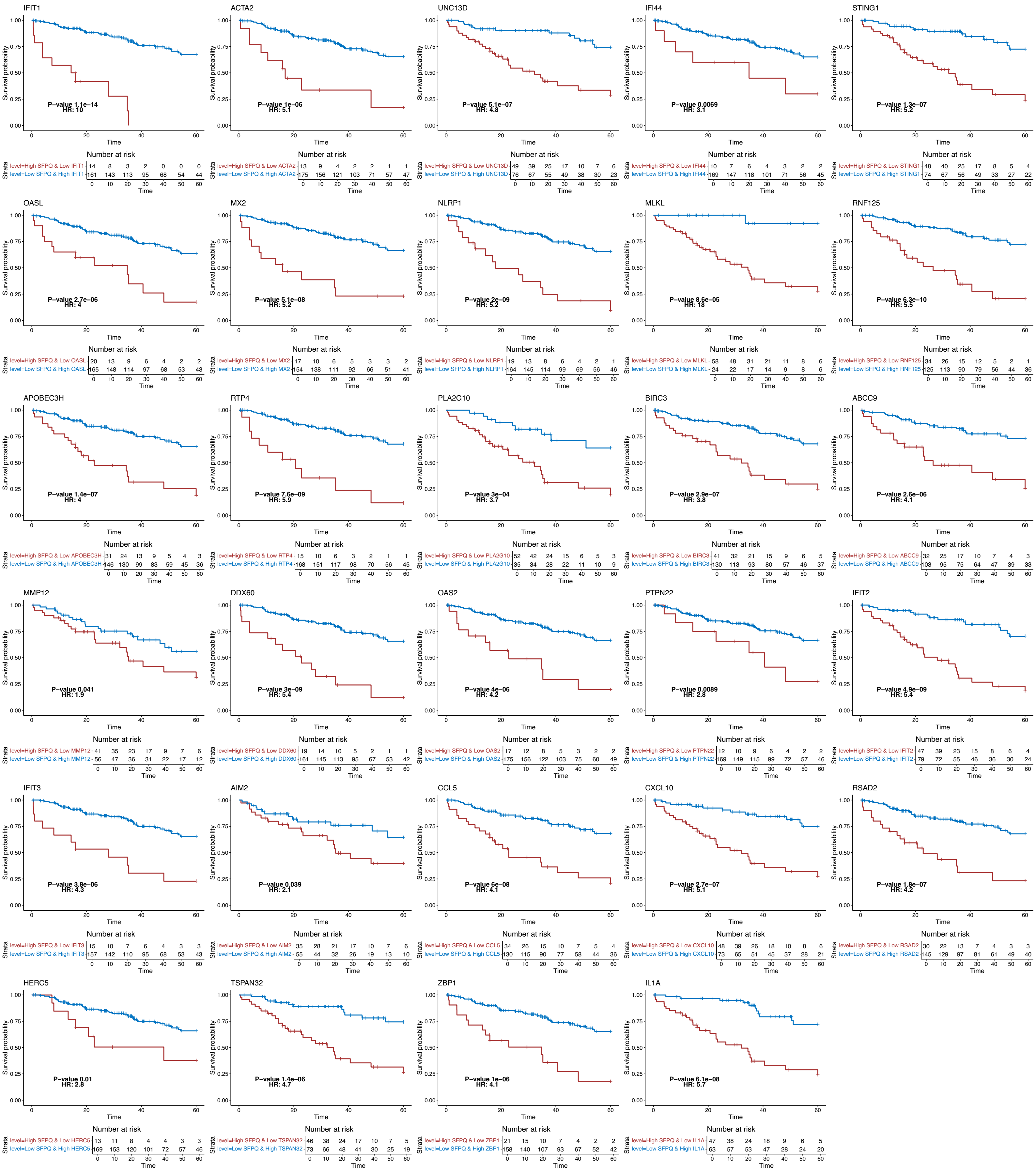

Survival probability
Time
Number at risk
Strata
IFIT1
P-value 1.1e−14
HR: 10
level=High SFPQ & Low IFIT1 14 8 3 2 0 0 0
level=Low SFPQ & High IFIT1 161 143 113 95 68 54 44
ACTA2
P-value 1e−06
HR: 5.1
level=High SFPQ & Low ACTA2 13 9 4 2 2 1 1
level=Low SFPQ & High ACTA2 175 156 121 103 71 57 47
UNC13D
P-value 5.1e−07
HR: 4.8
level=High SFPQ & Low UNC13D 49 39 25 17 10 7 6
level=Low SFPQ & High UNC13D 76 67 55 49 38 30 23
IFI44
P-value 0.0069
HR: 3.1
level=High SFPQ & Low IFI44 10 7 6 4 3 2 2
level=Low SFPQ & High IFI44 169 147 118 101 71 56 45
STING1
P-value 1.3e−07
HR: 5.2
level=High SFPQ & Low STING1 48 40 25 17 8 5 4
level=Low SFPQ & High STING1 74 67 56 49 33 27 22
OASL
P-value 2.7e−06
HR: 4
level=High SFPQ & Low OASL 20 13 9 6 4 2 2
level=Low SFPQ & High OASL 165 148 114 97 68 53 43
MX2
P-value 5.1e−08
HR: 5.2
level=High SFPQ & Low MX2 17 10 6 5 3 3 2
level=Low SFPQ & High MX2 154 138 111 92 66 51 41
NLRP1
P-value 2e−09
HR: 5.2
level=High SFPQ & Low NLRP1 19 13 8 6 4 2 1
level=Low SFPQ & High NLRP1 164 145 114 99 69 56 46
MLKL
P-value 8.6e−05
HR: 18
level=High SFPQ & Low MLKL 58 48 31 21 11 8 6
level=Low SFPQ & High MLKL 24 22 17 14 9 8 6
RNF125
P-value 6.3e−10
HR: 5.5
level=High SFPQ & Low RNF125 34 26 15 12 5 2 1
level=Low SFPQ & High RNF125 125 113 90 79 56 44 36
APOBEC3H
P-value 1.4e−07
HR: 4
level=High SFPQ & Low APOBEC3H 31 24 13 9 5 4 3
level=Low SFPQ & High APOBEC3H 146 130 99 83 59 45 36
RTP4
P-value 7.6e−09
HR: 5.9
level=High SFPQ & Low RTP4 15 10 6 3 2 1 1
level=Low SFPQ & High RTP4 168 151 117 98 70 56 45
PLA2G10
P-value 3e−04
HR: 3.7
level=High SFPQ & Low PLA2G10 52 42 24 15 6 5 3
level=Low SFPQ & High PLA2G10 35 34 28 22 11 10 9
BIRC3
P-value 2.9e−07
HR: 3.8
level=High SFPQ & Low BIRC3 41 32 21 15 9 6 5
level=Low SFPQ & High BIRC3 130 113 93 80 57 46 37
ABCC9
P-value 2.6e−06
HR: 4.1
level=High SFPQ & Low ABCC9 32 25 17 10 7 4 3
level=Low SFPQ & High ABCC9 103 95 75 64 47 39 33
MMP12
P-value 0.041
HR: 1.9
level=High SFPQ & Low MMP12 41 35 23 17 9 7 6
level=Low SFPQ & High MMP12 56 47 36 31 22 17 12
DDX60
P-value 3e−09
HR: 5.4
level=High SFPQ & Low DDX60 19 14 10 5 2 1 1
level=Low SFPQ & High DDX60 161 145 113 95 67 53 42
OAS2
P-value 4e−06
HR: 4.2
level=High SFPQ & Low OAS2 17 12 8 5 3 2 2
level=Low SFPQ & High OAS2 175 156 122 103 75 60 49
PTPN22
P-value 0.0089
HR: 2.8
level=High SFPQ & Low PTPN22 12 10 9 6 4 2 2
level=Low SFPQ & High PTPN22 169 149 115 99 72 57 46
IFIT2
P-value 4.9e−09
HR: 5.4
level=High SFPQ & Low IFIT2 47 39 23 15 8 6 4
level=Low SFPQ & High IFIT2 79 72 55 46 36 30 24
IFIT3
P-value 3.8e−06
HR: 4.3
level=High SFPQ & Low IFIT3 15 10 7 6 4 3 3
level=Low SFPQ & High IFIT3 157 142 110 95 68 53 43
AIM2
P-value 0.039
HR: 2.1
level=High SFPQ & Low AIM2 35 28 21 17 10 7 6
level=Low SFPQ & High AIM2 55 44 32 26 19 13 10
CCL5
P-value 6e−08
HR: 4.1
level=High SFPQ & Low CCL5 34 26 15 10 7 5 4
level=Low SFPQ & High CCL5 130 115 90 77 58 44 36
CXCL10
P-value 2.7e−07
HR: 5.1
level=High SFPQ & Low CXCL10 48 39 26 18 10 8 6
level=Low SFPQ & High CXCL10 73 65 51 45 37 28 21
RSAD2
P-value 1.8e−07
HR: 4.2
level=High SFPQ & Low RSAD2 30 22 13 7 4 3 3
level=Low SFPQ & High RSAD2 145 129 97 81 61 49 40
HERC5
P-value 0.01
HR: 2.8
level=High SFPQ & Low HERC5 13 11 8 4 4 3 3
level=Low SFPQ & High HERC5 169 153 120 101 72 57 46
TSPAN32
P-value 1.4e−06
HR: 4.7
level=High SFPQ & Low TSPAN32 46 38 24 17 10 7 5
level=Low SFPQ & High TSPAN32 73 66 48 41 30 25 19
ZBP1
P-value 1e−06
HR: 4.1
level=High SFPQ & Low ZBP1 21 15 10 7 4 2 2
level=Low SFPQ & High ZBP1 158 140 107 93 67 52 42
IL1A
P-value 6.1e−08
HR: 5.7
level=High SFPQ & Low IL1A 47 38 24 18 9 6 5
level=Low SFPQ & High IL1A 63 57 53 47 28 24 20

Supplementary Figure 9

Kaplan-Meier curves showing human sarcoma patient overall survival. Patients were stratified for high SFPQ expression combined with low expression of individual innate immunity signature genes (High SFPQ & Low Signature gene) or low SFPQ expression paired with concomitant high expression of individual innate immunity signature gene (Low SFPQ & High Signature gene). Signature genes are shown in Fig. 7b (*IFIT1, ACTA2, UNC13D, IFI44, STING1, OASL, MX2, NLRP1, MLKL, RNF125, APOBEC3H, RTP4, PLAG2G10, BIRC3, ABCC9, MMP12, DDX60, OAS2, PTPN22, IFIT2, IFIT3, AIM2, CCL5, CXCL10, RSAD2, HERC5, TSPAN32, ZBP1* and *IL1A*). Data was obtained from the TCGA human sarcoma dataset. HR, hazard ratio; p-values are shown.

Genes	Hazard Ratio	Cox p-value	Curve p-value
IFIT1	0,33	7,96714E-06	2,67369E-06
ACTA2	0,39	0,002000781	0,001348573
UNC13D	0,44	0,002440335	0,001841574
IFI44	0,62	0,133092185	0,129863035
STING1	0,45	0,00327299	0,00255384
OASL	0,56	0,028964038	0,026911402
MX2	0,46	0,001588507	0,001210675
NLRP1	0,38	9,47749E-05	4,98899E-05
MLKL	0,1	0,024248661	0,005641339
RNF125	0,38	2,13785E-05	1,02687E-05
APOBEC3H	0,53	0,004594883	0,003959534
RTP4	0,31	5,48798E-06	1,55978E-06
PLA2G10	0,69	0,245223767	0,241800774
BIRC3	0,47	0,000674768	0,000497186
ABCC9	0,57	0,014039285	0,012818809
MMP12	1,5	0,077323832	0,075508698
DDX60	0,41	0,000343743	0,000216458
OAS2	0,31	2,05849E-05	6,71604E-06
PTPN22	0,55	0,037546475	0,035058001
IFIT2	0,39	0,000613407	0,000383493
IFIT3	0,5	0,006489905	0,005501578
AIM2	1,5	0,106869681	0,104716246
CCL5	0,49	0,001499657	0,001194913
CXCL10	0,42	0,002039146	0,00147084
RSAD2	0,49	0,001891899	0,001515908
HERC5	0,49	0,014489297	0,012457109
TSPAN32	0,52	0,01327683	0,011676956
ZBP1	0,52	0,007947196	0,00693515
IL1A	0,43	0,003033951	0,002252034

Supplementary table 1:
Signature genes used for Kaplan-Meier analysis in Supplementary Figure 8

For each gene in the signature, survival association was assessed using a Cox proportional hazards regression model (coxph), with gene expression dichotomized into High vs Low groups (cutpoints automatically determined using surv_cutpoint). Hazard ratios (HRs) and corresponding two-sided Wald test P values are reported. In parallel, Kaplan-Meier survival curves were compared using a two-sided log-rank (Mantel-Cox) test, and the resulting p-values are shown. Survival times were administratively censored at 60 months. Unless otherwise stated, nominal p-values are reported and no multiple-comparison adjustment was applied.

Genes	Hazard Ratio	Cox p-value	Curve p-value
IFIT1	10	3,21161E-10	1,08625E-14
ACTA2	5,1	1,12685E-05	1,03729E-06
UNC13D	4,8	4,71427E-06	5,09283E-07
IFI44	3,1	0,01012175	0,006871674
STING1	5,2	1,78702E-06	1,33046E-07
OASL	4	1,41251E-05	2,7003E-06
MX2	5,2	9,88878E-07	5,08101E-08
NLRP1	5,2	7,30442E-08	2,01602E-09
MLKL	18	0,004471184	8,56785E-05
RNF125	5,5	3,07331E-08	6,26056E-10
APOBEC3H	4	1,03567E-06	1,36556E-07
RTP4	5,9	3,43473E-07	7,55301E-09
PLA2G10	3,7	0,000704387	0,000298946
BIRC3	3,8	1,71543E-06	2,88659E-07
ABCC9	4,1	1,37089E-05	2,55617E-06
MMP12	1,9	0,04455394	0,041021578
DDX60	5,4	1,16745E-07	2,99843E-09
OAS2	4,2	2,2216E-05	3,99467E-06
PTPN22	2,8	0,012278704	0,008909227
IFIT2	5,4	1,41978E-07	4,93325E-09
IFIT3	4,3	2,20086E-05	3,80746E-06
AIM2	2,1	0,044136418	0,039442714
CCL5	4,1	5,61442E-07	6,02353E-08
CXCL10	5,1	3,315E-06	2,68284E-07
RSAD2	4,2	1,48505E-06	1,83241E-07
HERC5	2,8	0,013556484	0,010019077
TSPAN32	4,7	1,11456E-05	1,40928E-06
ZBP1	4,1	6,5599E-06	1,0193E-06
IL1A	5,7	1,35056E-06	6,12375E-08

Supplementary table 2:
Signature genes used for Kaplan-Meier analysis in Supplementary Figure 9

For each gene in the signature, survival association was assessed using a Cox proportional hazards regression model (coxph), with gene expression dichotomized into High vs Low groups (cutpoints automatically determined using surv_cutpoint). Hazard ratios (HRs) and corresponding two-sided Wald test P values are reported. In parallel, Kaplan-Meier survival curves were compared using a two-sided log-rank (Mantel-Cox) test, and the resulting p-values are shown. Survival times were administratively censored at 60 months. Unless otherwise stated, nominal p-values are reported and no multiple-comparison adjustment was applied.

Target	Company	Cod/Sequence
Control-non target	Dharmacon	ON-TARGETplus Non-targeting Control siRNAs (D-001810-01-05)
Human SFPQ	Dharmacon	ON-TARGETplus Human SFPQ siRNA (L-006455-00-0005)
Human SFPQ 5'-UTR	Eurofins	5'- CCACGUUUCCUGAGCGUCU(dTdT)-3' This paper
Human SFPQ 5'UTR scramble	Eurofins	5' - GTTCTGCTGCCCGTCACTA (dTdT) – 3
Human DAXX	Eurofins	5'- GGAGUUGGAUCUCUCAGAA(dTdT)-3' Chen et al., 2003 [10]
Human RNaseH1	Eurofins	5'-ACAAACCAAAGAGCGGAAAUUCAUG(dTdT) -3' (Arora R. et al. 2014 [11])

Supplementary table 3: Synthetic siRNAs used in this study
