# Supplementary material for: SFPQ directs histone H3.3 deposition to R-loops in DNA repeats to protect genome stability
Source: Nat Commun. 2026 Feb 24;17:3151. doi: 10.1038/s41467-026-69479-w (PMC13043726; doi:10.1038/s41467-026-69479-w)
Supplement: Supplementary file 2 — Description of Additional Supplementary Files [file 41467_2026_69479_MOESM2_ESM.pdf]

Description of additional Supplementary files

File Name: Supplementary Data 1

Description: Sequences of EMSA probes used in this study

Sequences of the DNA and oligonucleotides used in the EMSA experiments are shown. Oligonucleotide length and their inclusion in specific EMSA substrates are indicated. [6FAM],

File Name: Supplementary Data 2

Description: Data of mass spectrometric analysis of protein band #1 shown in main figure 3A

Excel file reporting mass spectrometry data from SFPQ immunoprecipitates, including the peptides identified by mass spectrometry from band 1 shown in Fig. 3a of the manuscript. # Proteins, number of proteins identified in the sample or protein group; # Unique Peptides, peptides that map uniquely to a single protein, not shared with any other protein; # Peptides, total number of peptide sequences identified for that protein, including both unique and shared peptides; # PSMs (Peptide-Spectrum Matches), total number of MS/MS spectra that were matched to peptides belonging to that protein; # AAs, number of amino acids in the full-length protein sequence; MW 51 [kDa], molecular weight of the protein in kilodaltons, calculated from its amino acid sequence calc. pI, calculated isoelectric point.

File Name: Supplementary Data 3

Description: Data of mass spectrometric analysis of protein band #2 shown in main figure 3A

Excel file reporting mass spectrometry data from SFPQ immunoprecipitates, including the peptides identified by mass spectrometry from band 2 shown in Fig. 3a of the manuscript. # Proteins, number of proteins identified in the sample or protein group; # Unique Peptides, peptides that map uniquely to a single protein, not shared with any other protein; # Peptides, total number of peptide sequences identified for that protein, including both unique and shared peptides; # PSMs (Peptide-Spectrum Matches), total number of MS/MS spectra that were matched to peptides belonging to that protein; # AAs, number of amino acids in the full-length protein sequence; MW [kDa], molecular weight of the protein in kilodaltons, calculated from its amino acid sequence; calc. pI, calculated isoelectric point.

File Name: Supplementary Data 4

Description: Data of mass spectrometric analysis of protein band #3 shown in main figure 3A

Excel file reporting mass spectrometry data from SFPQ immunoprecipitates, including the peptides identified by mass spectrometry from band 3 shown in Fig. 3a of the manuscript. # Proteins, number of proteins identified in the sample or protein group; # Unique Peptides, peptides that map uniquely to a single protein, not shared with any other protein; # Peptides, total number of peptide sequences identified for that protein, including both unique and shared peptides; # PSMs (Peptide-Spectrum Matches), total number of MS/MS spectra that were matched to peptides belonging to that protein; # AAs, number of amino acids in the full-length protein sequence; MW [kDa], molecular weight of the protein in kilodaltons, calculated from its amino acid sequence; calc. pI, calculated isoelectric point.

File Name: Supplementary Data 5

Description: Data of mass spectrometric analysis of protein band #4 shown in main figure 3A

Excel file reporting mass spectrometry data from SFPQ immunoprecipitates, including the peptides 83 identified by mass spectrometry from band 4 shown in Fig. 3a of the manuscript. # Proteins, number of proteins identified in the sample or protein group; # Unique Peptides, peptides that map uniquely to a single protein, not shared with any other protein; # Peptides, total number of peptide sequences identified for that protein, including both unique and shared peptides; # PSMs (Peptide-Spectrum Matches), total number of MS/MS spectra that were matched to peptides belonging to that protein; # AAs, number of amino acids in the full-length protein sequence; MW [kDa], molecular weight of the protein in kilodaltons, calculated from its amino acid sequence; calc. pI, calculated isoelectric point.

File Name: Supplementary Data 6

Description: Data of mass spectrometric analysis of protein band #5 shown in main figure 3A

Excel file reporting mass spectrometry data from SFPQ immunoprecipitates, including the peptides identified by mass spectrometry from band 5 shown in Fig. 3a of the manuscript. # Proteins, number of proteins identified in the sample or protein group; # Unique Peptides, peptides that map uniquely to a single protein, not shared with any other protein; # Peptides, total number of peptide sequences identified for that protein, including both unique and shared peptides; # PSMs (Peptide-Spectrum Matches), total number of MS/MS spectra that were matched to peptides belonging to that protein; #

AAs, number of amino acids in the full-length protein sequence; MW [kDa], molecular weight of the protein in kilodaltons, calculated from its amino acid sequence; calc. pI, calculated

File Name: Supplementary Data 7

Description: Differentially expressed genes and functional annotation of RNA-seq data

Tables showing differentially expressed genes and the functional annotation of RNA-seq data obtained from U-2 OS cells subjected to transient, RNAi-mediated depletion of SFPQ. For methodological details, refer to the Methods section. Sheet 1, differential expression was assessed testing using Benjamini-Hochberg FDR. Genes passing FDR < 0.05 and $|\log2FC| \geq 1.5$ were classified as ABS, UP, or DW. Sheet2, enrichment p-values were computed using a one-sided Fisher's exact and Benjamini-Hochberg FDR correction

File Name: Supplementary Data 8

Description: Oligonucleotides

Table listing the PCR primers used in this study. Sequences, relevant literature references, and applications are indicated.

File Name: Supplementary Data 9

Description: Vectors used in this study.

Table indicates vectors used in this study. Addgene numbers, relevant literature references are indicated.

File Name: Supplementary Data 10

Description: Antibodies used in this study

Table listing the antibodies used in this study. The provider, product number, and antibody dilutions for different methodological applications are indicated. WB, western blotting; IF, immunofluorescence; ChIP or DRIP, chromatin immunoprecipitation or DNA:RNA immunoprecipitation; IP, immunoprecipitation.

File name: Supplementary Movie 1

Description: Live-cell microscopy showing mitotic defects in U-2 OS cells following RNAi-mediated depletion of SFPQ. The cells stably express GFP-tagged histone H2B. For methodological details, we refer to the Methods section. Movie refers to Fig. 6 of the manuscript.

127. Chen, L.-Y. & Chen, J. D. Daxx silencing sensitizes cells to multiple apoptotic pathways. *Mol Cell Biol* **23**, 7108–7121 (2003).

128. Miglietta, G., Russo, M., Duardo, R. C. & Capranico, G. G-quadruplex binders as cytostatic modulators of innate immune genes in cancer cells. *Nucleic Acids Res* **49**, 6673–6686 (2021).

129. Di Minin, G. *et al.* Mutant p53 Reprograms TNF Signaling in Cancer Cells through Interaction with the Tumor Suppressor DAB2IP. *Molecular Cell* **56**, 617–629 (2014).

130. Bates, R. C. & Mercurio, A. M. Tumor Necrosis Factor-α Stimulates the Epithelial-to-Mesenchymal Transition of Human Colonic Organoids. *MBoC* **14**, 1790–1800 (2003).

131. Abakir, A. *et al.* N6-methyladenosine regulates the stability of RNA:DNA hybrids in human cells. *Nat Genet* **52**, 48–55 (2020).

132. Tasselli, L. *et al.* SIRT6 deacetylates H3K18ac at pericentric chromatin to prevent mitotic errors and cellular senescence. *Nat Struct Mol Biol* **23**, 434–440 (2016).

133. Rosso, I. & d'Adda di Fagagna, F. Detection of Telomeric DNA:RNA Hybrids Using TeloDRIP-qPCR. *Int J Mol Sci* **21**, 9774 (2020).

134. Deng, Z. *et al.* A role for CTCF and cohesin in subtelomere chromatin organization, TERRA transcription, and telomere end protection. *EMBO J* **31**, 4165–4178 (2012).

135. Tsukahara, T., Haniu, H. & Matsuda, Y. PTB-Associated Splicing Factor (PSF) Is a PPARγ-Binding Protein and Growth Regulator of Colon Cancer Cells. *PLOS ONE* **8**, e58749 (2013).

136. Shastrula, P. K. *et al.* PML is recruited to heterochromatin during S phase and represses DAXX-mediated histone H3.3 chromatin assembly. *Journal of Cell Science* **132**, jcs220970 (2019).

137. Wu, H., Sun, H., Liang, X., Lima, W. F. & Crooke, S. T. Human RNase H1 Is Associated with Protein P32 and Is Involved in Mitochondrial Pre-rRNA Processing. *PLOS ONE* **8**, e71006 (2013).

138. Hou, H. *et al.* Expression and significance of cortactin and HDAC6 in human prostatic foamy gland carcinoma. *International Journal of Experimental Pathology* **96**, 248–254 (2015).

139. Rosonina, E. *et al.* Role for PSF in mediating transcriptional activator-dependent stimulation of pre-mRNA processing in vivo. *Mol Cell Biol* **25**, 6734–6746 (2005).

140. Enomoto, M., Bunge, M. B. & Tsoulfas, P. A multifunctional neurotrophin with reduced affinity to p75NTR enhances transplanted Schwann cell survival and axon growth after spinal cord injury. *Exp Neurol* **248**, 170–182 (2013).
