# Supplementary material for: SFPQ directs histone H3.3 deposition to R-loops in DNA repeats to protect genome stability
Source: Nat Commun. 2026 Feb 24;17:3151. doi: 10.1038/s41467-026-69479-w (PMC13043726; doi:10.1038/s41467-026-69479-w)
Supplement: Supplementary file 14 — Reporting Summary [file 41467_2026_69479_MOESM14_ESM.pdf]

nature portfolio

Corresponding author(s): Stefan Schoeftner

Last updated by author(s): 2026/01/08

Reporting Summary

Nature Portfolio wishes to improve the reproducibility of the work that we publish. This form provides structure for consistency and transparency in reporting. For further information on Nature Portfolio policies, see our Editorial Policies and the Editorial Policy Checklist.

Statistics

For all statistical analyses, confirm that the following items are present in the figure legend, table legend, main text, or Methods section.

n/a	Confirmed	
☐	☒	The exact sample size (*n*) for each experimental group/condition, given as a discrete number and unit of measurement
☐	☒	A statement on whether measurements were taken from distinct samples or whether the same sample was measured repeatedly
☐	☒	The statistical test(s) used AND whether they are one- or two-sided<br>*Only common tests should be described solely by name; describe more complex techniques in the Methods section.*
☒	☐	A description of all covariates tested
☒	☐	A description of any assumptions or corrections, such as tests of normality and adjustment for multiple comparisons
☐	☒	A full description of the statistical parameters including central tendency (e.g. means) or other basic estimates (e.g. regression coefficient) AND variation (e.g. standard deviation) or associated estimates of uncertainty (e.g. confidence intervals)
☐	☒	For null hypothesis testing, the test statistic (e.g. *F*, *t*, *r*) with confidence intervals, effect sizes, degrees of freedom and *P* value noted<br>*Give P values as exact values whenever suitable.*
☒	☐	For Bayesian analysis, information on the choice of priors and Markov chain Monte Carlo settings
☒	☐	For hierarchical and complex designs, identification of the appropriate level for tests and full reporting of outcomes
☒	☐	Estimates of effect sizes (e.g. Cohen's *d*, Pearson's *r*), indicating how they were calculated

Our web collection on statistics for biologists contains articles on many of the points above.

Software and code

Policy information about availability of computer code

Data collection	Cells subjected to immunofluorescence and DNA-FISH were captured with Leica Application Suite (LAS) imaging software. ChIP-qPCR, DRIP-qPCR, and RT-PCR data were coolected using CFX Maestro software.
Data analysis	ImageJ v.1.53e and Adobe Photoshop (2020) were used for image analysis and processing. Microsoft Excel and GraphPad Prism 8 were used for data analysis, statistical analysis, and graphs plotting.<br>RNA-seq and ChIP-seq: DESseq2 package inside R language framework for RNA-seq Analysis was used following standard procedure. ChIP-AP package (https://github.com/JSuryatenggara/ChIP-AP) and R language framework were used following standard procedure.

For manuscripts utilizing custom algorithms or software that are central to the research but not yet described in published literature, software must be made available to editors and reviewers. We strongly encourage code deposition in a community repository (e.g. GitHub). See the Nature Portfolio guidelines for submitting code & software for further information.

Data

Policy information about availability of data

All manuscripts must include a data availability statement. This statement should provide the following information, where applicable:

- Accession codes, unique identifiers, or web links for publicly available datasets
- A description of any restrictions on data availability
- For clinical datasets or third party data, please ensure that the statement adheres to our policy

Original data related to ChIP-Seq and RNA-Seq have been deposited at Gene Expression Omnibus (GEO) under the accession GSE281893 and GSE281892, respectively. Data is publicly available as of the date of publication. This study analyzed existing, publicly available data from the TCGA Research Network. Source data are provided with this paper.

Research involving human participants, their data, or biological material

Policy information about studies with human participants or human data. See also policy information about sex, gender (identity/presentation), and sexual orientation and race, ethnicity and racism.

Reporting on sex and gender	Sex and/or gender was not considered in the study design. Results are not specifically related to gender or sex.
Reporting on race, ethnicity, or other socially relevant groupings	The study does not report results with specific relevance for race, ethnicity or other social groups.
Population characteristics	Not applicable for the study
Recruitment	No patients were recruited by the authors of the study. Patient data was retrieved from the The Cancer Genome Atlas (TCGA)
Ethics oversight	Not applicable for the study

Note that full information on the approval of the study protocol must also be provided in the manuscript.

Field-specific reporting

Please select the one below that is the best fit for your research. If you are not sure, read the appropriate sections before making your selection.

☒ Life sciences ☐ Behavioural & social sciences ☐ Ecological, evolutionary & environmental sciences

For a reference copy of the document with all sections, see nature.com/documents/nr-reporting-summary-flat.pdf

Life sciences study design

All studies must disclose on these points even when the disclosure is negative.

Sample size	We did not perform a formal a priori sample size calculation. For experiments such as western blots, immunofluorescence analysis, ChIP, DRIP, qRT-PCR and genome instability analyses, we used 3–4 independent biological replicates per condition, which represent a standard sample size in this field and are widely considered sufficient to capture biologically meaningful differences while allowing robust statistical comparisons. Sample sizes were determined based on established practice in similar studies, our prior experience with variability in these assays, and practical constraints related to experimental complexity and resources. The reproducibility of the observed effects across independent biological replicates indicates that the sample sizes used were adequate to support the conclusions of the study. ChIP-seq experiments were performed as biological replicates; RNA-seq as biological triplicates. Details on sample numbers can be obtained from respective figures and figure legends. Statistical analysis (as described in respective figure legends) were used to calculate statistical significance of obtained results. The individual p-values are indicated in all figures.
Data exclusions	No data exclusion.
Replication	All experiments were performed a minimum of three times, except for ChIP-seq wich was performed twice. Number of biological replicates are specified in respective figures and figure legends.
Randomization	Pictures of immunofluorescence and DNA-FISH were randomly taken. All experiments based on gain or loss of function were performed with appropriate controls and repeated at least 3 times.
Blinding	Blinding was not performed because the experiments did not involve group allocation in a way that could introduce observer bias. Data collection and analysis were based on objective, quantitative readouts (e.g., biochemical assays, immunoblots, and molecular quantifications), which are not influenced by investigator expectations. For this reason, blinding was not relevant to the study design.

Reporting for specific materials, systems and methods

We require information from authors about some types of materials, experimental systems and methods used in many studies. Here, indicate whether each material, system or method listed is relevant to your study. If you are not sure if a list item applies to your research, read the appropriate section before selecting a response.

Materials & experimental systems

n/a	Involved in the study
☐	☒ Antibodies
☐	☒ Eukaryotic cell lines
☒	☐ Palaeontology and archaeology
☒	☐ Animals and other organisms
☒	☐ Clinical data
☐	☒ Dual use research of concern
☒	☐ Plants

Methods

n/a	Involved in the study
☐	☒ ChIP-seq
☒	☐ Flow cytometry
☒	☐ MRI-based neuroimaging

Antibodies

Antibodies used

Mouse anti-Actin (Sigma, Cat#A5441)
Rabbit anti-ATR (Cell Signaling Technology, Cat# 2790)
Rabbit anti-ATRX (Bethyl, Cat# A301-045A)
Rabbit anti-cGAS (ABclonal, Cat# A8335)
Human anti-centromere protein (CREST) (Antibodies Incorporation, Cat# 15-235)
Rabbit anti-DAXX (Bethyl, Cat# A301-353A)
Rabbit anti-H3 (Abcam, Cat# ab1791)
Rabbit anti-H3.3 (Millipore, Cat# 09-838)
Mouse anti-HA (Sigma-Aldrich, Cat# H9658)
Rabbit anti-FANCD2 (Novus, Cat# NB100-182)
Mouse anti-IKBα (Cell Signaling Technology, Cat# 4814)
Rabbit anti-IRF3 (ABclonal, Cat# A11373)
Mouse anti-c-Myc (Cell Signaling Technology, Cat# 2276)
Mouse anti-NF-κB p65 (Cell Signaling Technology, Cat# 6956)
Rabbit anti-ATR (phospho Thr1989) (GeneTex, Cat# GTX128145)
Mouse anti-phospho IKBα (Cell Signaling Technology, Cat# 2859)
Rabbit anti-Phospho-IRF3-S396 (ABclonal, Cat# AP1333)
Rabbit anti-Phospho-NF-κB p65 (Ser536) (Cell Signaling Technology, Cat# 3033)
Rabbit anti-Phospho-Stat3 (Tyr705) (Cell Signaling Technology, Cat# 9145)
Rabbit anti-Phospho RPA32 (S33) (Bethyl, Cat# A300-246A)
Rabbit anti-Phospho STING (S336) (ABclonal, Cat# AP1223)
Mouse anti-RAD51 (Novus, Cat# NB100-148)
Mouse anti-RPA2 (Novus, Cat# NB100-158)
Rabbit anti-SFPQ (Bethyl, Cat# A301-321A)
Mouse anti-STAT3 (Cell Signaling Technology, Cat# 9139)
Rabbit anti-STING (Cell Signaling Technology, Cat# 80231)
Mouse anti-DNA-RNA Hybrid (S9.6) (Kerafast, Cat# ENH001)
Rabbit anti-TRF1 (Santa Cruz Biotechnology, Cat# sc-6165-R)
Mouse anti-TRF2 (Millipore, Cat# 05-521)
Rabbit anti-Vinculin (Cell Signaling Technology, Cat# 13901)
Goat anti-Mouse Alexa Fluor 555 (Thermo Fisher Scientific, Cat# A-21422)
Goat anti-Mouse Alexa Fluor 488 (Thermo Fisher Scientific, Cat# A-11001)
Goat anti-Rabbit Alexa Fluor 555 (Thermo Fisher Scientific, Cat# A-21428)
Goat anti-Rabbit Alexa Fluor 488 (Thermo Fisher Scientific, Cat# A-11008)
Goat anti-Human Alexa Fluor 555 (Thermo Fisher Scientific, Cat# A-21433)
mouse-IgG-control (Santa Cruz Biotechnology, Cat# sc-2025)
rabbit-IgG-control (Santa Cruz Biotechnology, Cat# sc-2027)
Mouse anti-dsRNA 35I9 (Abcam, ab27156)
Mouse anti-HSP90 (Santa Cruz, SC13119)

Validation

The specificity of Rabbit anti-TRF1, Mouse anti-TRF2, Rabbit anti-ATR (phospho Thr1989), Rabbit anti-Phospho RPA32 (S33) and Mouse anti-DNA-RNA Hybrid (S9.6) were previously validated by Petti et al. "SFPQ and NONO suppress RNA:DNA-hybrid-related telomere instability" (doi : 10.1038/s41467-019-08863-1). Mouse anti-c-Myc and Mouse anti-HA specificity was confirmed by on cells overexpressing tagged proteins (Fig. 5c). Rabbit anti-ATRX specificity was confirmed by staining on U-2 OS cells (ATRX negative, data not shown). Rabbit anti-STING and Rabbit anti-Phospho STING (S336) specificity was confirmed by WesternBlot analysis on cells treated with H151 STING inhibitor (Suppl. Fig. 7i,7l). Rabbit anti-ATRX specificity was confirmed by Gulve et al. (doi: 10.1038/s41467-022-32680-8). Rabbit anti-H3 specificity was confirmed by Lee at al. (doi: 10.1101/gad.350989.123). Rabbit anti-H3.3 specificity has been stated on the manufacturer's website (Millipore). Rabbit anti-FANCD2 specificity was confirmed by Sridharan et al. (doi: 10.1242/jcs.00294). Mouse anti-IKBα and Mouse anti-phospho IKBα specificity was validated by Zhong, et. al. (doi: 10.1080/15384047.2024.2403197). Rabbit anti-IRF3 pecificity has been stated on the manufacturer's website (ABclonal). Mouse anti-

STAT3 and Rabbit anti-Phospho-Stat3 (Tyr705) specificity was confirmed by Liu et al. (doi: 10.26508/lsa.202402831). Mouse anti-NF-κB p65 specificity was validated by Jing, et. al.(doi: 10.1080/0886022X.2024.2368090). Rabbit anti-Phospho-NF-κB p65 (Ser536) speicifity was validated by Chen, et. al. (doi: 10.1016/j.bioactmat.2024.10.016). Mouse anti-RAD51 specificity was validated by Ito et al. (doi: 10.1038/s41467-023-42576-w). Specificity of mouse anti-dsRNA 35I9 (Abcam, ab27156) is documanted at the provider's webpapge. Specificity of the mouse anti-HSP90 (Santa Cruz, SC13119) is documented on the provider's webpage. Mouse anti-RPA2 specificity has been stated on the manufaturer's website (Novus). Rabbit anti-Vinculin specificity was confirmed by Bishop, et. al. (doi: 10.3324/haematol.2024.285587). mouse-IgG-control and rabbit-IgG-control specificity has been stated on the manufaturer's website (Santa Crutz).

Eukaryotic cell lines

Policy information about cell lines and Sex and Gender in Research

Cell line source(s)

Cell lines were obtained by ATCC. U-2 OS cells (osteosarcoma, ATCC HTB-96); H1299 cells (carcinoma; non–small cell lung cancer, ATCC CRL-5803); MCF-10A (breast epithelial, ATCC CRL-10317); MCF-7 cells (ATCC HTB-22); HEK293 (human embryonic kidney, ATCC CRL-1573

Authentication

Human cell lines used were obtained from ATCC and have not been cultured for longer than 2 months, minimizing the risk of cross-contamination or genetic drift. Based on the nature of the experiments and the short culture duration, authentication was not deemed necessary

Mycoplasma contamination

All cell lines used in the study were tested negative for Mycoplasma contaminations by DAPI staining and PCR analysis.

Commonly misidentified lines
(See ICLAC register)

No commonly misidentified lines were used in the study.

Plants

Seed stocks

Not applicable

Novel plant genotypes

Not applicable

Authentication

Not applicable

ChIP-seq

Data deposition

☒ Confirm that both raw and final processed data have been deposited in a public database such as GEO.

☒ Confirm that you have deposited or provided access to graph files (e.g. BED files) for the called peaks.

Data access links
May remain private before publication.

https://www.ncbi.nlm.nih.gov/geo/query/acc.cgi?acc=GSE281893 ChIP-SEQ data
https://www.ncbi.nlm.nih.gov/geo/query/acc.cgi?acc=GSE281892 RNA.SEQ data

Files in database submission

GSM8631907 SiC_DAXX_rep1
GSM8631908 SiC_DAXX_rep2
GSM8631909 SiC_H3-3_rep1
GSM8631910 SiC_H3-3_rep2
GSM8631911 SiC_INP_rep1
GSM8631912 SiC_INP_rep2
GSM8631913 SiC_SFPQ_rep1
GSM8631914 SiC_SFPQ_rep2
GSM8631915 SiS_DAXX_rep1
GSM8631916 SiS_DAXX_rep2
GSM8631917 SiS_H3-3_rep1
GSM8631918 SiS_H3-3_rep2
GSM8631919 SiS_INP_rep1
GSM8631920 SiS_INP_rep2
GSM8631907 SiC_DAXX_rep1
GSM8631908 SiC_DAXX_rep2
GSM8631909 SiC_H3-3_rep1
GSM8631910 SiC_H3-3_rep2
GSM8631911 SiC_INP_rep1
GSM8631912 SiC_INP_rep2
GSM8631913 SiC_SFPQ_rep1

GSM8631914 SiC_SFPQ_rep2
GSM8631915 SiS_DAXX_rep1
GSM8631916 SiS_DAXX_rep2
GSM8631917 SiS_H3-3_rep1
GSM8631918 SiS_H3-3_rep2
GSM8631919 SiS_INP_rep1
GSM8631920 SiS_INP_rep2

Genome browser session (e.g. UCSC)	Genome Browser: ENSEMBL with ENSEMBL annotation v104 GTF

Methodology

Replicates	ChIP-seq experiment was performed in duplicate.
Sequencing depth	For RNA-seq, an average of 54.7M aligned reads for each sample was used.<br>For ChIP-seq, an average of 8M aligned reads for each sample was used.
Antibodies	Rabbit anti-DAXX, Bethyl<br>Rabbit anti-H3.3, Millipore<br>Rabbit anti-SFPQ, Bethyl
Peak calling parameters	ChIP-AP package (https://github.com/JSuryatenggara/ChIP-AP) tools was used with standard parameter. This tool uses Genrich MACS2 SICER2, HOMER using standard parameter for broad peaks calling.
Data quality	RNA-seq quality control performed with FastQC<br>ChIP-seq analysis was performed with the ChiP-AP pipeline, performing quality control (FastQC), read trimming and filtering (Bbduk and Trimmomatic).
Software	For RNA-seq: RNA-STAR aligner for alignment. DESeq2 for differential expression analysis. Gprofiler2 for functional annotation. Visualization procedure were performed using ggpubr, enrichplot. Survaval Analysis was performed using survminer and contsurvplot. ChIP-SEQ: BWA aligner for alignment, Bbduk and Trimmomatic for quality filtering. Visualitations were performed using ggpubr, webr, chIPpeakAnno.
